# Molecular Profiling and Tumor Biomarker Analysis of GOG281/LOGS: A Positive Late-Phase Trial of Trametinib for Recurrent/Persistent Low-Grade Serous Ovarian Carcinoma

**DOI:** 10.1158/1078-0432.CCR-25-3042

**Published:** 2025-12-11

**Authors:** Robert L. Hollis, Austin Miller, Heather A. Lankes, Kwong-Kwok Wong, William Rodgers, David Millan, Karen Carty, Robert L. Coleman, Kathleen N. Moore, Angeles Alvarez Secord, David M. O’Malley, John K. Chan, Andrea R. Hagemann, Stephanie Gaillard, Saketh R. Guntupalli, Mitchell I. Edelson, Peter G. Rose, Oliver Dorigo, Susana Banerjee, Ailith Ewing, Michael Churchman, Anil K. Sood, C. Simon Herrington, Charlie Gourley, David M. Gershenson

**Affiliations:** 1 The Nicola Murray Centre for Ovarian Cancer Research, Cancer Research UK Scotland Centre, Institute of Genetics and Cancer, University of Edinburgh, Edinburgh, United Kingdom.; 2 NRG Oncology, Clinical Trial Development Division, Biostatistics & Bioinformatics, Roswell Park Comprehensive Cancer Center, Buffalo, New York.; 3 NRG Oncology, Operations Center-Philadelphia East, Philadelphia, Pennsylvania.; 4 Division of Gynecologic Oncology, Department of Obstetrics and Gynecology, The Ohio State University Wexner Medical Center, Columbus, Ohio.; 5 Department of Gynecologic Oncology and Reproductive Medicine, The University of Texas MD Anderson Cancer Center, Houston, Texas.; 6 NYPQ, Department of Pathology, Weill Medical College of Cornell University, New York, New York.; 7 Queen Elizabeth University Hospital, Glasgow, United Kingdom.; 8 Cancer Research UK Clinical Trials Unit, Institute of Cancer Sciences, University of Glasgow, Glasgow, United Kingdom.; 9 Stephenson Cancer Center at the University of Oklahoma, Oklahoma City, Oklahoma.; 10 Duke Cancer Institute, Durham, North Carolina.; 11 Ohio State University and the James Cancer Center, Columbus, Ohio.; 12 Gynecologic Oncology, Sutter Cancer Research Consortium, San Francisco, California.; 13 Washington University, Gynecologic Oncology, St. Louis, Missouri.; 14 Sidney Kimmel Comprehensive Cancer Center, Johns Hopkins Sidney Kimmel Cancer Center, Baltimore, Maryland.; 15 Gynecologic Oncology, University of Colorado School of Medicine, UC Health CU Cancer Center, Anschutz Medical Campus, Aurora, Colorado.; 16 Abington Hospital, Gynecologic Oncology, Abington, Pennsylvania.; 17 Gynecologic Oncology, Case Comprehensive Cancer Center and Cleveland Clinic, Cleveland, Ohio.; 18 Division of Gynecologic Oncology, Department of Obstetrics and Gynecology, Stanford Women’s Cancer Center, Stanford Cancer Institute, Stanford, California.; 19 The Royal Marsden Hospital NHS Foundation Trust, The Institute of Cancer Research, London, United Kingdom.; 20 MRC Human Genetics Unit & CRUK Scotland Centre, Institute of Genetics and Cancer, The University of Edinburgh, Western General Hospital, Edinburgh, United Kingdom.

## Abstract

**Purpose:**

Low-grade serous ovarian carcinoma (LGSOC) is a distinct form of ovarian cancer characterized by younger patient age and relative chemoresistance. The GOG281/LOGS trial (NCT02101788) investigated the efficacy of the MEK inhibitor trametinib compared with physician’s choice standard-of-care (SOC) in patients with LGSOC with persistent/recurrent disease. The study demonstrated significantly improved progression-free survival (PFS) in the trametinib-treated arm.

**Experimental Design:**

Two hundred and sixty patients with recurrent/persistent LGSOC were enrolled and randomly assigned in GOG281. We performed molecular analysis of 170 patients with available tumor specimens, comprising whole-exome sequencing and phospho-ERK (pERK) IHC, to identify biomarkers of clinical benefit from trametinib. The demographics of the translational cohort (*n* = 170) were comparable with those of the total trial cohort.

**Results:**

High tumor pERK expression (greater than the median histoscore of 140) was associated with significantly prolonged PFS with trametinib treatment versus SOC (median 20.1 vs. 5.6 months, log-rank *P* < 0.0001; test for interaction *P* = 0.023). Tumors harboring canonical RAS–RAF–MAPK mutations (*KRAS/BRAF/NRAS*: 44/134, 32.8% of cases) had a higher response rate to trametinib (50.0% vs. 8.3%; Barnard’s *P* = 0.0004; test for interaction *P* = 0.054), but *KRAS*/*BRAF*/*NRAS* status was not predictive of prolonged PFS (test for interaction *P* = 0.719). *KRAS* amplification (*n* = 5 without *KRAS*/*NRAS*/*BRAF* mutation) and mutation of MAPK-associated genes (*n* = 25 without *KRAS*/*NRAS*/*BRAF* mutation or *KRAS* copy number gain) expanded the number of cases with identifiable MAPK defects to 55.2%, but consideration of these events did not improve the discrimination of trametinib responders. Chr1p loss (49% of cases) was associated with lower pERK expression (*P* = 0.021).

**Conclusions:**

This exploratory analysis suggests that pERK expression and mutation of *KRAS*/*BRAF*/*NRAS* are candidate biomarkers of improved PFS and response to trametinib, respectively.

**Translational Relevance**

GOG281 is the first positive late-phase trial in low-grade serous ovarian carcinoma (LGSOC), demonstrating improved progression-free survival (PFS) with trametinib use for recurrent/persistent LGSOC, in which standard-of-care response rates are low. However, it is clear that not all patients benefit from trametinib and that MEK inhibition is associated with specific additional toxicities. Biomarkers of response are therefore required to better define patient groups most likely to benefit. In this study, we perform molecular analysis of tumor specimens from individuals enrolled in GOG281 to identify such biomarkers. We identify *KRAS*/*BRAF*/*NRAS* mutation and high phospho-ERK expression to be associated with trametinib response and PFS benefit, respectively. Mutations affecting other MAPK pathway components occur frequently, but consideration of these defects did not improve the resolution of distinguishing trametinib responders from nonresponders. Together, these findings significantly advance our understanding of patients with LGSOC who do and do not benefit from MEK inhibitor therapy.

## Introduction

Low-grade serous ovarian carcinoma (LGSOC) is a unique form of ovarian, fallopian tube, or peritoneal cancer, accounting for around 5% of diagnoses (1, 2). LGSOC is characterized by younger patient age at diagnosis (median 46–48 years; ref. 3), frequent advanced stage at diagnosis (4), and marked survival benefit associated with complete macroscopic resection at first-line cytoreduction (5). LGSOC demonstrates relatively high levels of intrinsic chemotherapy resistance [objective response rate (ORR) ≤25% to first-line platinum-based regimens; ref. 6] compared with other forms of ovarian cancer, and treatment of persistent and recurrent disease is therefore a major clinical challenge.

LGSOC demonstrates frequent mutational activation of the RAS/RAF MAPK pathway, with approximately 50% of cases harboring activating mutations in *KRAS*, *BRAF*, or *NRAS* (1). Within the last 5 years, a number of studies have performed comprehensive genomic analysis of LGSOC samples from unselected cohorts to improve our understanding of genomic drivers beyond canonical MAPK mutations in this disease (7–11). These studies have demonstrated several additional recurrent genomic events in LGSOC, including *USP9X* mutation (∼10%–25% of cases), *EIF1AX* mutation (5%–10% cases), and chromosome 1 copy number alterations (in ∼50% of cases; refs. 7–11). These investigations have also identified relationships between specific events and clinicopathologic features; canonical MAPK pathway mutations (*KRAS*, *BRAF*, and *NRAS*) have been associated with prolonged survival (7, 9, 10, 12, 13), older age at diagnosis (7, 9, 10, 13), and macropapillary stromal invasion (14). Conversely, chr1p loss/chr1q gain has been associated with shorter survival time (9), desmoplastic stromal reaction, and low progesterone receptor expression (14).

In recent years, accumulating efficacy data have led to changes in recommendations for LGSOC management. Endocrine therapy demonstrates significant activity for disease control in LGSOC (15), is well tolerated, and is now recommended for use as either first-line adjuvant therapy or first-line maintenance (16), though no randomized late-phase trial data are currently available in this disease context. The NRG-GY-019 phase III trial is currently ongoing to compare first-line adjuvant letrozole monotherapy versus conventional chemotherapy (carboplatin plus paclitaxel) and subsequent letrozole maintenance in LGSOC (17).

The phase III MILO/ENGOT-ov11 study of the MEK inhibitor binimetinib versus physician’s choice chemotherapy (pegylated liposomal doxorubicin, paclitaxel, or topotecan) for recurrent/persistent LGSOC met the predefined progression-free survival (PFS) HR for futility in an interim analysis but demonstrated an ORR of 16% to binimetinib (18). GOG281/LOGS investigated the MEK inhibitor trametinib in recurrent/persistent LGSOC, demonstrating an ORR of 26% and significantly improved PFS compared with physician’s choice standard-of-care (SOC; pegylated liposomal doxorubicin, paclitaxel, topotecan, letrozole, or tamoxifen; HR = 0.48; ref. 19). Together, these late-phase studies have led to the recommendation of MEK inhibitors (trametinib or binimetinib) as treatment options for persistent/recurrent LGSOC (16). More recently, the RAF/MEK clamp avutometinib has shown notable efficacy when combined with the FAK inhibitor defactinib for recurrent LGSOC, with a response rate of 31% (20); this combination is now approved by the FDA for *KRAS*-mutant relapsed LGSOC following reporting of a 44% response rate in the *KRAS*-mutant subpopulation. However, it is clear that not all patients derive benefit from MEK-targeted therapy; some groups of patients respond less frequently [*KRAS*, *BRAF*, and *NRAS* wild-type (WT) cases], and treatment resistance can develop (18, 19). Moreover, treatment with these agents is frequently associated with significant toxicity, further highlighting the need for selection of patients most likely to derive substantial clinical benefit.

Improved understanding of the molecular landscape of LGSOC that subsequently recurs represents an opportunity to identify further events that may be therapeutically exploitable. Moreover, comprehensive characterization of samples from MEK inhibitor–treated patients affords the opportunity to identify biomarkers of response, better informing selection of patients who are most likely to benefit from these regimens. Here, we report comprehensive genomic characterization of tumor specimens from patients enrolled in the GOG281/LOGS trial of trametinib, the first positive late-phase trial in LGSOC (19). We integrate these data with quantified phospho-ERK (pERK) expression levels to investigate molecular features that may be associated with trametinib response and outcomes in the context of recurrent LGSOC.

## Materials and Methods

### GOG281 patient cohort

GOG281/LOGS was an international 1:1 randomized open-label phase II/III clinical trial evaluating the efficacy of trametinib versus investigators’ choice SOC (oral letrozole 2·5 mg once daily; oral tamoxifen 20 mg twice daily; intravenous paclitaxel 80 mg per m^2^ body surface area on days 1, 8, and 15 of every 28-day cycle; intravenous pegylated liposomal doxorubicin 40–50 mg per m^2^ body surface area once every 4 weeks; or intravenous topotecan 4 mg per m^2^ body surface area on days 1, 8, and 15 of every 28-day cycle) in recurrent/persistent LGSOC across the United Kingdom and United States (NCT02101788); for full protocol details, see ref. 19. Archival formalin-fixed paraffin-embedded (FFPE) samples from 189 patients who later enrolled in GOG281 were available for translational analysis (19). Samples were archival material from diagnosis. Objective response and investigator-assessed PFS were evaluated according to RECIST version 1.1. The study was performed in accordance with the Declaration of Helsinki. All participants gave written informed consent. The study received Institutional Review Board approval by the NCI and the East of Scotland Research Ethics Service Research Ethics Committee 2.

### Whole-exome sequencing

**Figure 1.**
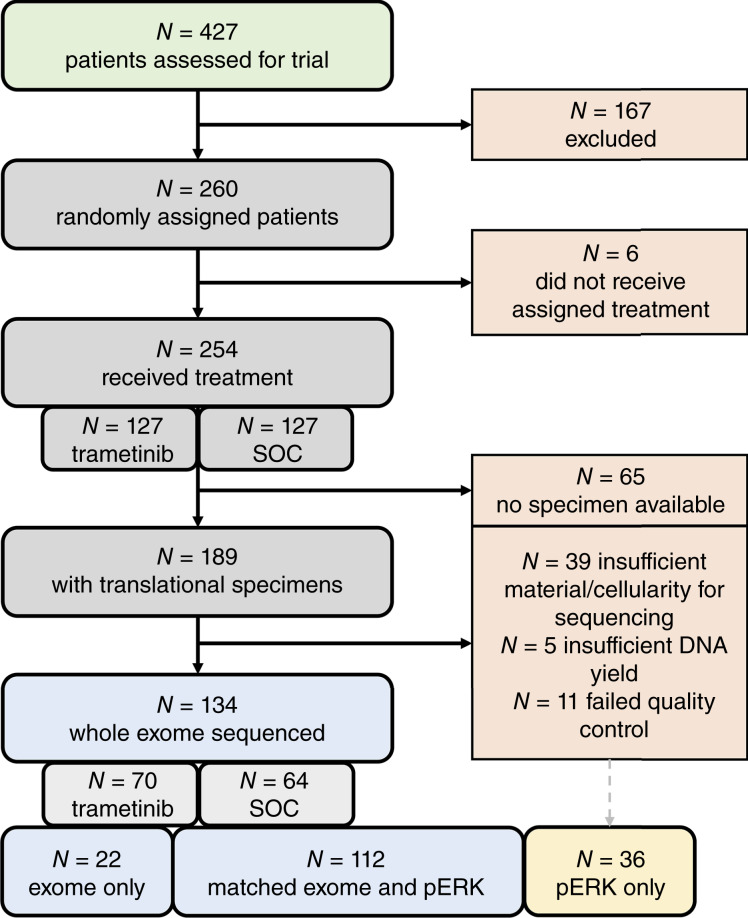
Study case flow diagram for WES of translational specimens from patients enrolled in GOG281. One hundred thirty-four samples underwent successful WES, of which 112 were also evaluable for pERK via IHC. Additionally, 36 samples were evaluable for pERK IHC that did not have matching WES.

Of the 189 cases with translational specimens (Fig. 1), 150 had samples of sufficient cellularity for genomic analysis (≥40% achievable tumor cellularity upon macrodissection, based upon pathologic review). FFPE material was macrodissected using hematoxylin and eosin–stained slides, marked to identify areas of invasive carcinoma by an expert gynecologic pathologist (CSH), prior to DNA extraction.

Genomic profiling by whole-exome sequencing (WES) was performed using the Agilent SureSelect Human All Exon V6 Exome Capture Kit and Illumina NovaSeq 6000, as described previously (19). Reads were aligned to GRCh38 and variants called within the bcbio-nextgen high-throughput sequencing pipeline using a majority vote system from three variant callers (Freebayes, Mutect2, and VarDict). Five cases failed DNA quality control (DNA yield <200 ng) and were excluded prior to sequencing. Eleven samples failed sequencing quality control (*n* = 10 excessive FFPE damage, *n* = 1 <50× mean target coverage), leaving 134 samples with WES. The median per-sample on-target coverage achieved in the cohort was 102× (range, 59–172×). Mutations identified in *KRAS*, *BRAF*, and *NRAS* have been previously reported (19).

Called variants were annotated using the Ensembl variant effect predictor and filtered to exclude common variation, variants at low variant allele frequency (VAF < 0.1), variants with low alternate read depth (<8 reads), known benign variants, silent changes, and noncoding variation. Remaining variants of known pathogenicity from the ClinVar database (RRID: SCR_006169) and high-impact variants (nonsense mutations, frameshifting mutations, translational start site changes, splice site mutations, and changes affecting stop codons) were flagged as functional. Remaining missense changes that were predicted to be damaging by both PolyPhen (RRID: SCR_013189) and SIFT (RRID: SCR_012813) tools were retained as potentially functional. Other variants (splice region variants, in-frame changes, missense variants not predicted deleterious by either SIFT, or PolyPhen) were excluded as variants of unknown significance.

MAPK-associated genes were defined as those within the Gene Ontology Term GO:0000165 (“MAPK cascade”), retrieved using biomaRt (RRID: SCR_019214; ref. 21).

### Copy-number variation

Genome-wide copy-number estimates of 30 kb genomic intervals across the genome were calculated using CopywriteR (RRID: SCR_025864; ref. 22), with aligned BAM files from the bcbio variant calling pipeline used as an input, from which chromosome arm-level copy-number changes were identified. To detect chromosome arm-level events, the median relative log_2_ copy-number ratios of intervals spanning each chromosome arm were calculated. To automatically detect chromosome arm-level copy-number aberration, medians of 0.3 and −0.3 were used as thresholds for gains and losses, respectively. This automated approach was compared with manual assessment of visualized copy number calculated across the 30 kb segments within the p and q arms of chr1-6 to identify gains and losses (Supplementary Fig. S1). The sensitivity and specificity of the automated detection compared with manual curation were 94.6% and 98.1%, respectively.

To identify clusters of tumors defined by chromosome arm-level copy-number events, loss/gain calls were translated into a copy-number matrix of called gain/loss events across samples (loss, −1; gain, 1; neutral, 0). This matrix underwent hierarchical clustering using Ward’s linkage and Euclidean distance to identify clusters of tumor samples based upon patterns of gain and loss.

### pERK expression analysis

pERK expression was assessed by IHC using 5 µm tumor FFPE sections available for 154 cases, of which 148 were evaluable (*n* = 6 no evaluable area of definitive carcinoma). IHC was performed using anti–phospho-p44/42 ERK1/2 rabbit mAb (Cell Signaling Technology, #4370S, 1:300 dilution; RRID: AB_2315112) on the BondMax Autostainer within the CLIA-certified MD Anderson Cancer Center Clinical Immunohistopathology Laboratory. pERK levels were quantified manually (KKW) using whole-cell histoscore (Supplementary Fig. S2; refs. 23, 24), whereby the percentage of positive tumor cells (0%–100%) was multiplied by expression intensity (0, negative; 1, weak; 2, intermediate; and 3, strong) to generate a quantified numeric measure of expression from 0 to 300. A second independent observer (CSH) was used to validate the generated scores, demonstrating strong agreement (rho = 0.85, *P* < 0.0001; Supplementary Fig. S2).

### Statistical analysis

Predictive biomarker analysis was performed using SAS version 9.4, utilizing multivariable Cox proportional hazards and logistic regression models with covariate adjustment for stratification factors as described previously (19). The Cox regression model used to assess pERK as a predictive biomarker for PFS included main effects for treatment (trametinib vs. SOC) and pERK histoscore (stratified by median histoscore; >140 vs. ≤140), as well as their interaction term. Stratification by the median pERK histoscore was adopted due to the absence of a clear distribution-suggested cutoff (Supplementary Fig. S2) and the reported 50% frequency of canonical RAS–RAF MAPK activating mutations in this tumor type (2). Performance status and prior lines of therapy were stratified. The prediction *P* value was estimated as the type 3 test for the interaction term. Time to progression was characterized using Kaplan–Meier methods, with corresponding *P* values estimated by stratified log-rank tests. Biomarkers to predict overall response were assessed using multivariable logistic regression models specified as described above. All assessment and assignment of molecular subgroups were performed blinded to outcome data.

All other statistical analyses were performed using R version 4.2.2. Comparisons of frequency were made using Barnard’s unconditional test. Correlation analysis was performed using Spearman’s rank correlation coefficient. Comparisons of continuous data were made using the Mann–Whitney U test. All tests were two-sided, unless otherwise specified. Nominal *P* values less than 0.05 were considered statistically significant.

This study was performed and reported in line with the REMARK EQUATOR framework.

## Results

### GOG281 translational cohort

The translational analysis cohort comprised 170 cases with suitable diagnostic FFPE tumor material that underwent molecular analysis [*n* = 112 with both WES and pERK expression analysis (Fig. 1); *n* = 36 with pERK analysis only; *n* = 22 with WES only]. The demographics of the translational cohort were highly comparable with the total GOG281 clinical trial cohort (Table 1; Supplementary Table S1).

**Table 1.** Demographics of translational research population.

	Translational cohort					
	All, *N* = 170		Arm			
			SOC, *N* = 85		Trametinib, *N* = 85	
	*N*	%	*N*	%	*N*	%
Age (years)	​	​	​	​	​	​
18–29	15	8.8	7	8.2	8	9.4
30–39	19	11.2	12	14.1	7	8.2
40–49	33	19.4	18	21.2	15	17.6
50–59	42	24.7	17	20.0	25	29.4
60–69	43	25.3	23	27.1	20	23.5
≥70	18	10.6	8	9.4	10	5.9
Performance status	​	​	​	​	​	​
ECOG 0	122	71.8	63	74.1	59	69.4
ECOG 1	48	28.2	22	25.9	26	30.6
Stage at diagnosis	​	​	​	​	​	​
FIGO I/II	24	14.1	10	11.8	14	16.5
FIGO III	129	75.9	65	76.5	64	75.3
FIGO IV	17	10.0	10	11.8	7	8.2
Primary site	​	​	​	​	​	​
Ovary	153	90.0	76	89.4	77	90.6
Peritoneum	17	10.0	9	10.6	8	9.4
Best response	​	​	​	​	​	​
CR	2	1.2	1	1.2	1	1.2
PR	21	12.4	4	4.7	17	20.0
Stable	113	66.5	60	70.6	53	62.4
Progressing	23	13.5	17	20.0	6	7.1
Undetermined	11	6.5	3	3.5	8	9.4
Survival status	​	​	​	​	​	​
Alive	93	54.7	43	50.6	50	58.8
Deceased	77	45.3	42	49.4	35	41.2

Abbreviations: CR, complete response; ECOG, Eastern Cooperative Oncology Group; FIGO, International Federation of Gynecology and Obstetrics; PR, partial response.

### pERK expression and trametinib outcome

**Figure 2.**
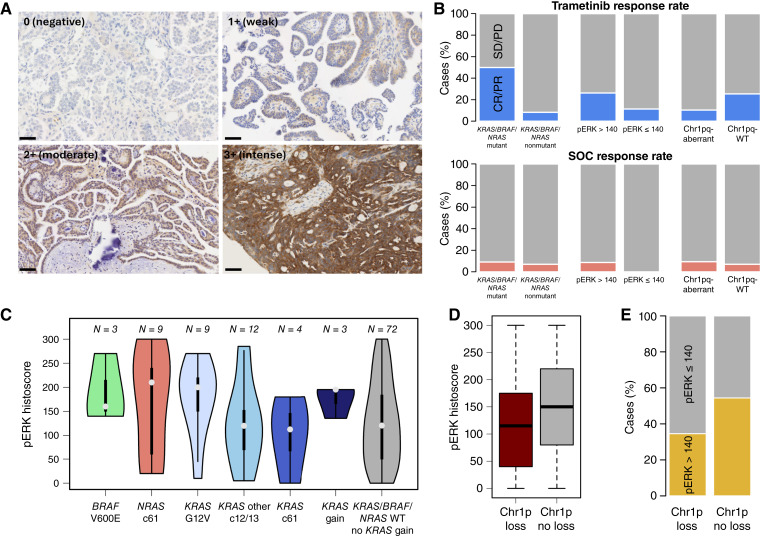
pERK and other potential biomarkers of therapy response in LGSOC. **A,** pERK IHC examples demonstrating negative (0), weak (1+), moderate (2+), and intense (3+) positivity for histoscore calculation. Scale bars, 50 µm. **B,** Response to trametinib (top) and physician’s choice SOC (bottom) according to *KRAS*/*BRAF*/*NRAS* mutation status (left), pERK status (middle), and chr1 abnormalities (right). **C,** pERK histoscore according to *KRAS*/*BRAF*/*NRAS* mutation type. Labels specify the numbers within each group that were evaluable for pERK expression levels. **D,** pERK histoscore by chr1p-loss status. **E,** Frequency of pERK status between chr1p-loss and -intact groups. Chr1pq-aberrant, chr1p-loss with concurrent chr1q-gain.

**Figure 3.**
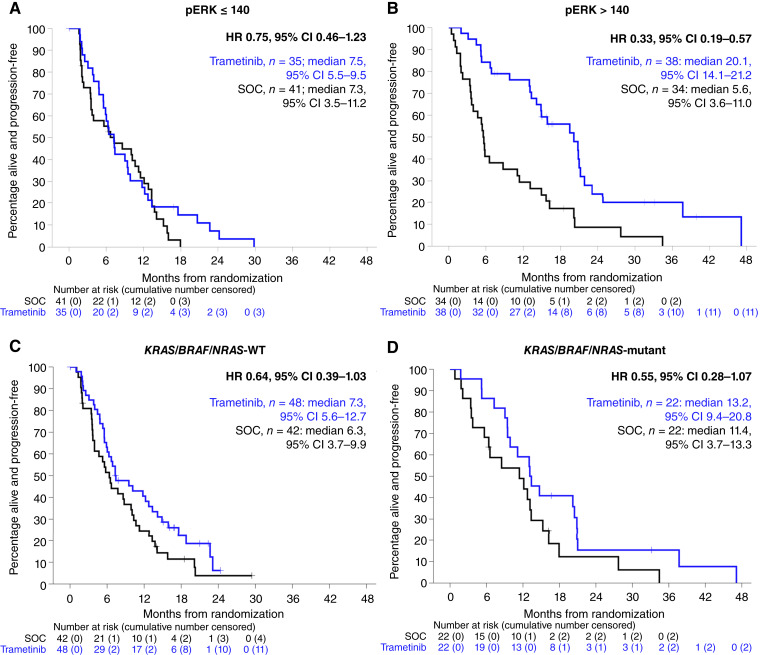
Impact of pERK status and *KRAS*/*BRAF*/*NRAS* mutation status on GOG281 patient outcome. **A,** PFS of GOG281/LOGS patients with low-pERK tumors (pERK ≤ 140) in trametinib versus SOC arms. **B,** PFS of GOG281/LOGS patients with high-pERK tumors (pERK > 140) in trametinib versus SOC arms. **C,** PFS of GOG281/LOGS patients with *KRAS*/*BRAF*/*NRAS* WT tumors in trametinib versus SOC arms. **D,** PFS of GOG281/LOGS patients with *KRAS*/*BRAF*/*NRAS*-mutant tumors in trametinib versus SOC arms. Labeled HR refer to comparison of trametinib versus SOC arm.

pERK expression status was available for 148 evaluable cases (Fig. 2; Supplementary Tables S1 and S2; Supplementary Fig. S3). The median pERK histoscore across cases was 140 (range, 0–300; Fig. 2A; Supplementary Fig. S2), with pERK staining demonstrating relatively low heterogeneity across whole sections (Supplementary Fig. S3). In cases with high pERK expression, defined by a pERK histoscore greater than the median (histoscore >140), trametinib treatment was associated with significantly prolonged PFS (median 20.1 vs. 5.6 months in SOC arm, log-rank *P* < 0.0001), whereas those with low pERK expression (histoscore ≤140) demonstrated no significant difference in PFS time between each arm (7.2 months in trametinib-treated vs. 7.3 in SOC-treated; Fig. 3). The interaction between pERK expression status, treatment, and PFS was statistically significant (test for interaction *P* = 0.023), and the observed pERK-high subgroup-specific PFS benefit was tolerant to adjusting the threshold for defining high pERK expression across the 120 to 160 histoscore range (Supplementary Table S3).

The response rate to trametinib and SOC in patients with high pERK tumors was 35.7% (10/28 evaluable cases) and 8.8% (3/34 cases), respectively. In patients with low pERK tumors (histoscore ≤140), the response rate to trametinib and SOC was 11.4% (4/35 cases) and 0% (0/41 cases), respectively. Although a significantly greater proportion of patients with high-pERK tumors demonstrated an objective response to trametinib compared with those with low-pERK tumors (35.7%, 10/28 vs. 11.4%, 4/35, Barnard’s *P* = 0.023; Fig. 2B), pERK expression status was not predictive of response (test for interaction *P* = 0.464).

When comparing trametinib-treated patients against those who received chemotherapy SOC specifically (paclitaxel, liposomal doxorubicin, or topotecan), the benefit of trametinib in the high-pERK subgroup remained substantial [HR for trametinib vs chemotherapy SOC 0.23, 95% confidence interval (CI), 0.09–0.56; median PFS in the trametinib-treated group 15.9 months, 95% CI, 13.0–21.0; median PFS in the chemotherapy-treated group 5.4 months, 95% CI, 1.9–11.4]. Trametinib treatment was not associated with a significant survival benefit in those with low-pERK tumors (median PFS in the trametinib-treated group 6.8 months, 95% CI, 2.5–9.5; median PFS in the chemotherapy-treated group 10.2 months, 95% CI, 3.5–13.3; Supplementary Fig. S4).

### Genomic features of the GOG281 cohort

**Figure 4.**
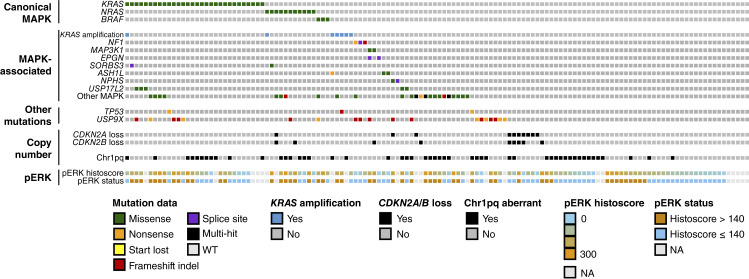
Molecular landscape of tumor samples from patients enrolled in GOG281/LOGS. MAPK-associated, MAPK-associated genes as defined in the Gene Ontology Term GO:0000165; Chr1pq-aberrant, chr1p-loss with concurrent chr1q-gain.

Of 134 evaluable cases, 44 (32.8%) demonstrated canonical MAPK mutations (30 *KRAS*, 11 *NRAS*, and three *BRAF*) as previously reported (19), all of which were missense mutations (Fig. 4). Twenty-three *KRAS* mutations were at codon 12 (10 G12D, 9 G12V, three G12C, and one G12S), whereas five were at codon 61 (two Q61K and three Q61H); the remaining two were at codon 13 (both G13D). All *NRAS* mutations were at codon 61 (10 Q61R and one Q61K). All three *BRAF* mutations were V600E. As anticipated, a greater proportion of *KRAS*/*BRAF*/*NRAS* patients had a pERK histoscore >140 (Barnard’s one-sided *P* = 0.038); however, the difference in proportions between *KRAS*/*NRAS*/*BRAF* mutant and WT was modest (pERK > 140: 57%, 21/37 evaluable mutant vs. 39%, 29/75 evaluable WT). Tumors with *BRAF*-V600E and *KRAS*-G12V mutations consistently showed high pERK levels (Fig. 2C). Three *KRAS*/*BRAF*/*NRAS*-WT cases with *KRAS* gain were evaluable for pERK expression and also showed relatively high pERK expression (Fig. 2C).

Seven cases demonstrated evidence of *KRAS* amplification (absolute copy number ≥6), five of which were *KRAS*/*NRAS*/*BRAF*-WT (Fig. 4). Forty cases demonstrated one or more mutations across other MAPK-associated genes defined within the MAPK Gene Ontology Term GO:0000165, including *NF1* and *MAPK3K1* (Fig. 4; Supplementary Table S4). Three cases had *NF1* mutation, and two cases had *MAPK3K1* mutation; none of these co-occurred with *KRAS*/*BRAF*/*NRAS* mutation (Fig. 4). In total, 25 cases demonstrated MAPK-associated mutation without *KRAS* amplification or *KRAS*/*BRAF*/*NRAS* mutation. Accordingly, the frequency of patients with aberrations across all the MAPK pathway (*KRAS*/*BRAF*/*NRAS* mutation, *KRAS* amplification, or MAPK-associated mutation) was 55.2%, comprising 44 *KRAS*/*BRAF*/*NRAS*-mutant, five *KRAS* gain (without *KRAS*/*BRAF*/*NRAS* mutation), and 25 with MAPK-associated gene mutation in the absence of other MAPK defects (Fig. 4).

In addition, 14.2% of cases demonstrated *USP9X* mutation, whereas *TP53* mutation frequency was low (2.2%: two nonsense mutations and one frameshift indel; Fig. 4). Here, 7.5% of cases demonstrated *CDKN2A* copy-number loss (absolute copy number <1, representing at least heterozygous loss). A pathogenic *BRCA1*/*2* variant was detected in a single case (*BRCA2* c.755_758del *p*.Asp252fs, rs80359659, VAF 0.45); this case was *TP53* WT and *KRAS*/*BRAF*/*NRAS* WT.

**Figure 5.**
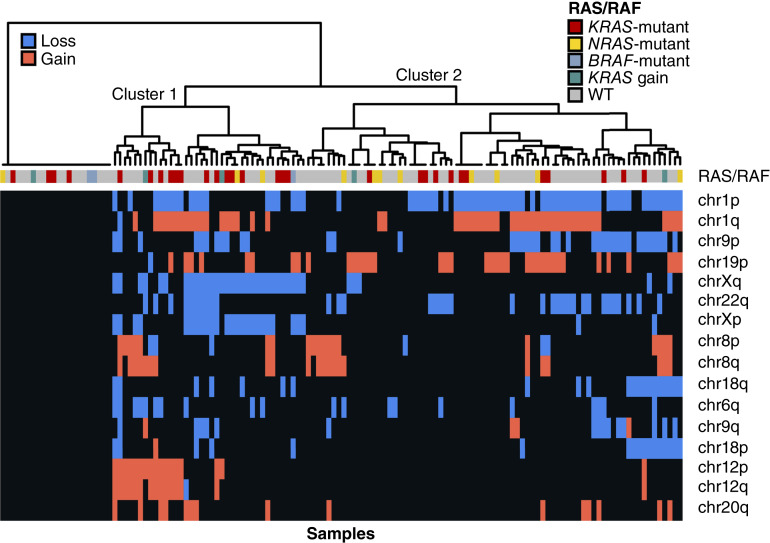
Unsupervised hierarchical clustering of chromosome arm-level copy-number alterations across biospecimens from patients enrolled in GOG281/LOGS.

Chr1p loss and chr1q gain were common events (49% and 38% of cases, respectively), with 30% of the cohort demonstrating concurrent chr1p-loss/chr1q-gain (chr1pq-aberrant; *P* < 0.0001 for enrichment of co-occurrence; Fig. 4). Copy-number aberrations of chr9p, chr19p, and chrX were also common (Fig. 5). Clustering of samples based on chromosome arm-level copy-number events highlighted a copy-number cluster with apparent enrichment of *KRAS* mutation (36.8% in cluster 1 vs. 16.7% in others; Barnard’s *P* = 0.022), whereas *NRAS* mutations predominantly occurred within the other major cluster (8 of 11, 72.7% of *NRAS* mutations within cluster 2; Fig. 5).

### MAPK-associated events and trametinib outcome

As previously reported (19), tumor *KRAS*/*BRAF*/*NRAS* mutations were associated with higher response rate to trametinib (trametinib response 50% in *KRAS*/*BRAF*/*NRAS*-mutant, 11/22 cases vs. 8.3%, 4/48 in *KRAS*/*BRAF*/*NRAS*-WT; Barnard’s *P* = 0.0004. SOC response 9.1% in *KRAS*/*BRAF*/*NRAS*-mutant, 2/22 cases vs. 7.1%, 3/42 cases in *KRAS*/*BRAF*/*NRAS*-WT. Test for interaction *P* = 0.054; Fig. 2B). Of the 15 genomically characterized cases that demonstrated complete or partial response to trametinib, five were *KRAS*-mutant, four were *NRAS*-mutant, two were *BRAF*-mutant, and four were *KRAS*/*BRAF*/*NRAS*-WT. The genotype-specific response rate to trametinib in *KRAS*-, *NRAS*-, and *BRAF*-mutant cases was 31.8% (5/16 trametinib-treated *KRAS*-mutant cases), 100% (4/4 trametinib-treated *NRAS*-mutant cases), and 100% (2/2 trametinib-treated *BRAF*-mutant cases). The response rate in the *KRAS*/*NRAS*/*BRAF*-WT cases that received trametinib was 8.3% (4 of 48 cases).

*KRAS*/*BRAF*/*NRAS* mutation status was not predictive of improved PFS on trametinib treatment (test for interaction *P* = 0.719). Expansion of the MAPK-aberrant population to include patients with tumors demonstrating *KRAS* amplification or MAPK-associated gene mutations did not improve the discrimination between responders and noresponders (MAPK-aberrant: trametinib response 33.3%, 13/39 cases; SOC response 11.4%, 4/35 cases. MAPK WT: trametinib response 6.5%, 2/31 cases; SOC response 3.4%, 1/29 cases).

The response rate to trametinib in cases with both *KRAS*/*BRAF*/*NRAS* mutation and high pERK expression was 50% (6/12 trametinib-treated cases with both pERK > 140 and *KRAS*/*BRAF*/*NRAS* mutation).

### Chr1pq events and treatment outcome

The response rate in cases with chr1pq-aberrant tumors was low in both the trametinib (10.5%, 2/19 evaluable cases) and SOC (9.5%, 2/21) treatment arms (Fig. 2B). Although the trametinib response rate in patients with chr1pq-aberrant tumors was lower than in the remaining population (10.5% vs. 25.5%, 13 of 51 evaluable cases), the difference did not approach statistical significance (Barnard’s *P* = 0.118). The response rate to SOC in patients with tumors without concurrent chr1p-loss/chr1q-gain was 7.0% (3 of 43 evaluable cases). The median PFS in the chr1pq-aberrant population was 9.5 and 5.7 months in the trametinib- and SOC-treated arms, respectively. In cases without concurrent chr1p-loss/chr1q-gain, the median PFS was 11.8 and 7.7 months in the trametinib- and SOC-treated arms, respectively.

The frequency of *KRAS*/*BRAF*/*NRAS* mutation was similar in tumors with and without chr1p-loss/chr1q-gain (30.0%, 12/40% vs. 34.0%, 32/94). Chr1p-loss specifically was associated with significantly lower pERK expression (median histoscore 115 vs. 150, *P* = 0.021; Fig. 2D), with significantly fewer chr1p-loss tumors demonstrating a pERK histoscore >140 (34.5%, 19/55 pERK > 140 in chr1p-loss vs. 54.4%, 31/57 in chr1p-WT; Barnard’s *P* = 0.036; Fig. 2E).

## Discussion

Over the last 5 years, substantial advances have been made in our understanding of LGSOC (1, 2). These include the recognition of endocrine therapy and MEK-targeted agents as clinically effective treatment options (15, 18, 20), alongside a greater understanding of molecular events present in these tumors (7–11). Molecular profiling of clinical trial samples represents a key opportunity for association of recurrent molecular events with treatment response and outcome, leveraging the high-quality clinical annotation associated with patients enrolled on late-phase studies. GOG281/LOGS was an international (United Kingdom and United States) phase II/III study of trametinib for recurrent/persistent LGSOC which met its primary endpoint of improved PFS in the trametinib-treated arm versus SOC (19). Here, we present molecular characterization of tumor samples from patients enrolled in GOG281/LOGS, the first positive late-phase trial of patients with LGSOC.

We identify high levels of pERK as a putative predictor of improved PFS with trametinib treatment. Patients with tumors demonstrating a pERK histoscore greater than the median (histoscore >140) experienced a 14.5-month higher median PFS with trametinib compared with SOC (median PFS 20.1 vs. 5.6 months; HR = 0.33, 95% CI, 0.33–0.57). ERK is downstream of MEK in the RAS/RAF MAPK pathway, providing a readout of pathway activity levels (25). High pERK levels are indicative of high MEK activation; this high activity provides a rationale for why higher pERK levels are associated with improved benefit from trametinib, which directly inhibits MEK. Although pERK status was associated with a difference in PFS between trametinib versus SOC, we did not identify any differences in response rates based upon pERK status. This suggests that trametinib may have a cytostatic rather than a cytotoxic effect in pERK-high tumors.

Given that IHC is a universally applied tool in contemporary diagnostic pathology, and that histoscore is a widely used approach for quantifying expression intensity (23), pERK IHC represents a readily implementable method by which to identify patients most likely to derive PFS benefit from MEK inhibition. As this assay requires very little input material, application to biopsies from disease relapse is wholly feasible to capture the pERK status specifically in recurrent disease, rather than relying on historic samples from diagnosis, which may precede relapse by several years. The findings from our study alone do not suggest a specific order in which *KRAS*/*BRAF*/*NRAS* mutational testing versus pERK IHC should be performed. However, the high response rate to trametinib in patients with *KRAS*/*BRAF*/*NRAS*-mutant tumors combined with the relative ease of interpreting somatic variants in these genes may argue for mutational testing in the first instance, followed by pERK IHC in WT samples.

Although many samples in our study demonstrated relatively uniform pERK staining across whole slides, we did observe a degree of intratumor heterogeneity. Moreover, LGSOC often harbors a substantive borderline tumor component, making interpretation of stained slides more complex. Although IHC is a readily implementable methodology, numerous scoring strategies exist for quantifying expression; we did not investigate the relative utility of different scoring methods, nor did we seek to standardize or optimize a specific threshold for defining the pERK-high population. Substantial inter-observer variability has been demonstrated in quantifying clinically relevant IHC biomarkers, even when specifically comparing scores from expert pathologists (26). A full evaluation of inter-observer variability, optimal scoring methodology, and establishment of an optimal threshold for defining high-pERK tumors is warranted before such a test can be considered for clinical implementation.

Within the primary analysis of GOG281/LOGS, patients with tumors harboring *KRAS*/*BRAF*/*NRAS* mutation demonstrated higher trametinib response rates (50% vs. 8.3% for *KRAS*/*BRAF*/*NRAS* WT), though this failed to achieve significance upon formal test for interaction (*P* = 0.054; ref. 19). In the phase II RAMP-201 trial, the response rate to avutometinib–defactinib combination therapy was 44% in the *KRAS*-mutant population and 17% in the *KRAS*-WT population; accordingly, the subsequent FDA approval for this combination is limited to the *KRAS*-mutant subpopulation (20). These findings were recapitulated in *post hoc* analysis of cases from the MILO study of the MEK inhibitor binimetinib, with a response rate of 41% in patients with tumors harboring mutation in *KRAS*, *BRAF*, *NRAS*, or *NF1* (27); the test for interaction between *KRAS*/*BRAF*/*NRAS*/*NF1* status and response did not approach significance.

Identification of *NF1* mutation in tumors from patients in the MILO study highlights the potential importance of MAPK pathway–activating genomic events beyond canonical *KRAS*, *BRAF*, and *NRAS* mutations (27). Through WES, we investigated the frequency of mutation in a large number of genes associated with the MAPK pathway. We demonstrate that a large proportion of *KRAS*/*BRAS*/*NRAS*-WT tumors harbor mutations in other MAPK-associated genes. Together with *KRAS* amplification, these defects expanded the MAPK-aberrant population from 32.8% to 55.2%. However, inclusion of these additional cases in analysis of trametinib response did not improve the resolution of responders versus nonresponders in the GOG281/LOGS population. These data suggest that not all the identified defects have a substantive impact on MAPK activity; the biological significance of candidate pathogenic variants in the majority of these genes is yet to be established. In particular, it is unclear which are potential driver events and which are passengers. Caution is therefore warranted when including tumors with such variants within the MAPK genomically activated subtype of LGSOC. Notably, the response rate of the remaining MAPK WT population was extremely low in both the trametinib-treated (6.5%) and SOC (3.4%) arms.

As RAS/RAF inhibition is a shared mechanism of action for avutometinib, trametinib, and binimetinib, pERK status and *KRAS*/*BRAF*/*NRAS* status are also likely to be informative regarding avutometinib response rate. ENGOTov60/GOG-3052/RAMP-201 reported a 44% ORR to avutometinib plus defactinib among the 57 patients with *KRAS*-mutated tumors, compared with 17% in the 52 patients with *KRAS*-WT tumors (28). Similarly, we anticipate that pERK status is likely to be informative in the context of binimetinib treatment. The low response rate to trametinib, binimetinib, and avutometinib in *KRAS*/*NRAS*/*BRAF*-WT cases across all available studies further underscores the need for additional treatment strategies in LGSOC, and recent studies have highlighted several additional agents for investigation (1). These include the CDK4/6 inhibitors abemaciclib and ribociclib (29, 30), and the kinase inhibitor dasatinib (31), alongside other SRC family kinase inhibitors (31).

In unselected LGSOC populations, the frequency of *KRAS*, *BRAF*, and *NRAS* mutation is collectively around 50%, with 33%, 10%, and 10% of cases demonstrating mutations in each of these genes, respectively (7–11). Within the present GOG281 cohort, which is selected for recurrent/persistent disease, we identified these events in only 33% of cases. This may be explained by the reports of improved survival in patients with LGSOC with tumors harboring *KRAS*, *BRAF*, or *NRAS* mutation (7, 9, 10), leading to a depletion of this molecular subgroup in cohorts selected for relapse. Moreover, *BRAF* mutation has been more frequently associated with serous borderline tumors than invasive LGSOC (32); LGSOC demonstrating *BRAF* mutation may manifest a less aggressive phenotype with more indolent behavior, leading to further depletion of these cases at relapse. Indeed, the frequency of *BRAF* mutation specifically in our cohort was only 2%.

Using chromosome arm-level copy-number status, we also identify distinct clusters of LGSOC tumor samples based upon patterns of gains and losses. One of these clusters was significantly enriched for *KRAS*-mutant tumors, suggesting associations between *KRAS* mutation status and the broader genomic landscape in LGSOC. Chromosome arm-level copy-number changes at chr1 are common in LGSOC, with 30% showing concurrent chr1p-loss and chr1q-gain. Although Chr1pq aberration is not predictive of response or PFS benefit from trametinib, cases with chr1p-loss demonstrated lower pERK expression, suggesting a potential association between genomic copy-number events and MAPK activity levels in LGSOC.

Together, our findings identify high tumor pERK levels as a marker of improved PFS with trametinib treatment in recurrent/persistent LGSOC, whereas *KRAS*/*BRAF*/*NRAS* mutation is associated with higher response rate to trametinib. Expanding the definition of MAPK-aberrant to genes associated with the wider MAPK pathway reveals a substantive population with candidate MAPK-associated defects in tumors that would otherwise have been considered MAPK WT. However, this expansion does not result in the identification of a genomic MAPK defect in all trametinib responders, suggesting mechanisms beyond short mutational events that are able to drive therapeutically exploitable RAS/RAF MAPK pathway activity in LGSOC.

## Supplementary Material

Supplementary Data 1 Supplementary Data

## Data Availability

Participant data for individuals enrolled in GOG281/LOGS, including a data dictionary defining each field in the set, are available on www.clinicaltrials.gov. Requests for deidentified patient-level data from studies funded through the NCI Cancer Therapy Evaluation Program must comply with the US Department of Health and Human Services and Office for Human Research Protections policies and requirements. Requests for sharing of deidentified patient-level data should be sent to the GOG281/LOGS lead investigators (David Gershenson and Charlie Gourley) and will be considered on a case-by-case basis with the NCI Cancer Therapy Evaluation Program. Raw sequencing data were generated in a previous study (19). Processed sequencing data (variant calls) are available in the supplement of this article, alongside chromosome arm-level copy-number calls and pERK expression data.
