# Supplementary material for: Molecular Profiling and Tumor Biomarker Analysis of GOG281/LOGS: A Positive Late-Phase Trial of Trametinib for Recurrent/Persistent Low-Grade Serous Ovarian Carcinoma
Source: Clin Cancer Res. 2025 Dec 11;32(4):724–34. doi: 10.1158/1078-0432.CCR-25-3042 (PMC13056244; doi:10.1158/1078-0432.CCR-25-3042)
Supplement: Supplementary Data 1 — Supplementary Data [file ccr-25-3042_supplementary_data_1_suppsd_1.pdf]

Molecular profiling and tumour biomarker analysis of GOG281/LOGS: a positive late-phase trial of trametinib for recurrent/persistent low grade-serous ovarian carcinoma

Supplementary figures

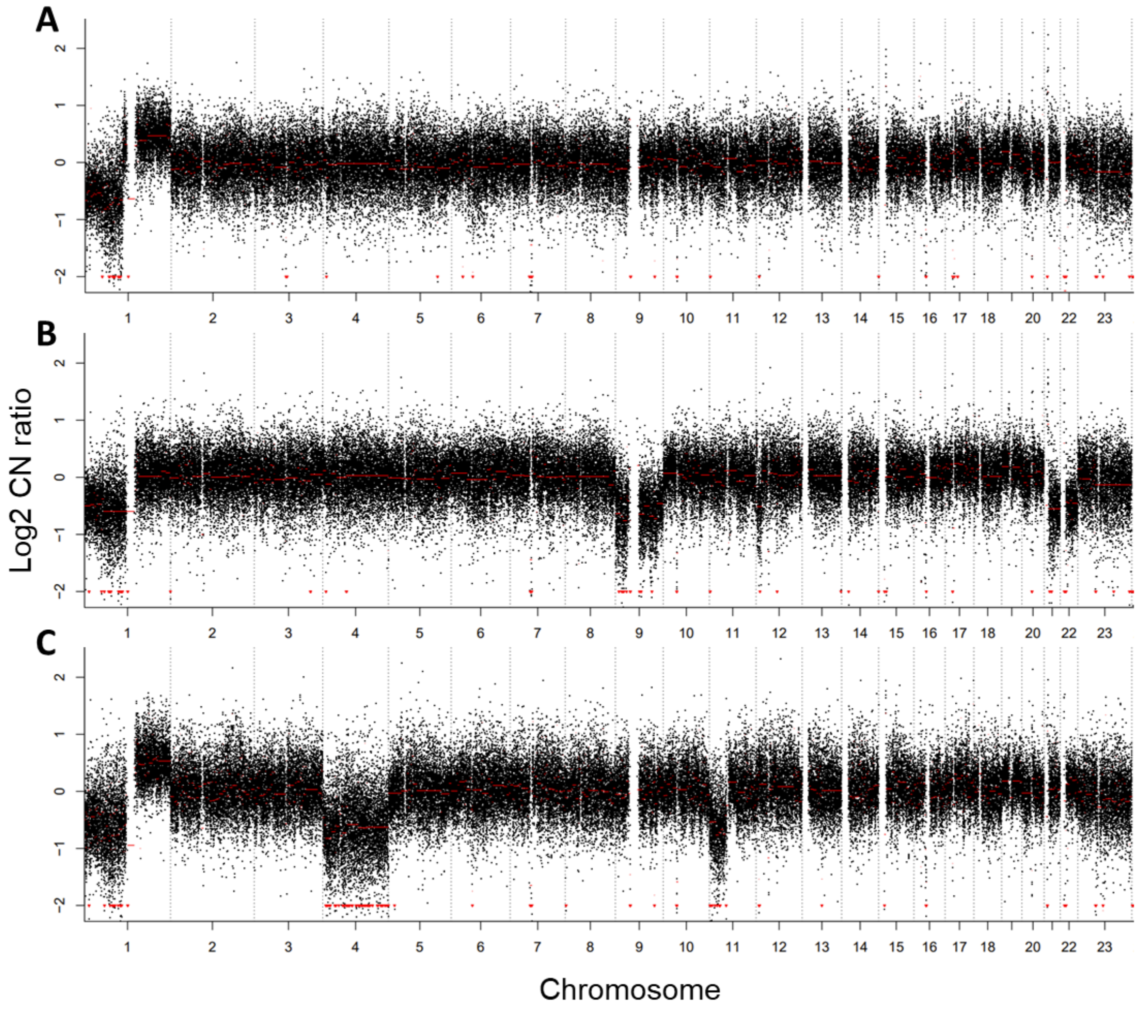

Supplementary Figure S1. Visualised copy number across 30kB genomic intervals in LGSOC samples. (A) LGSOC sample showing concurrent Chr1p loss and Chr1q gain, with few other copy number aberrations. (B) LGSOC sample with Chr1p loss without concurrent Chr1q gain, alongside other aberrations on chromosome 9 and others. (C) LGSOC sample showing concurrent Chr1p loss and Chr1q gain, alongside other loss events on chromosome 4 and others.

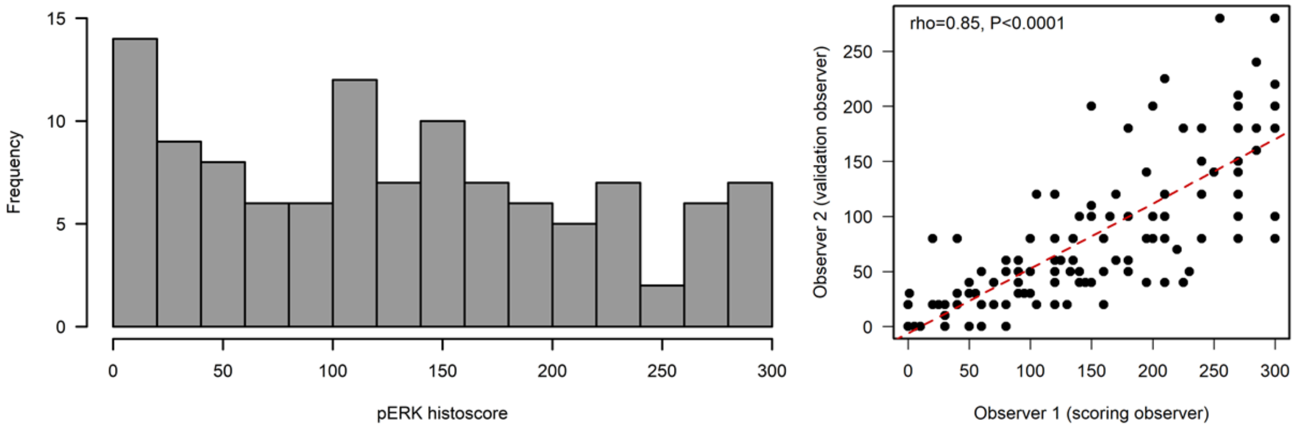

Supplementary Figure S2. Phospho-ERK (pERK) histoscoring. Left: histogram of pERK expression quantified by histoscore; right: validation of pERK histoscoring with a second observer.

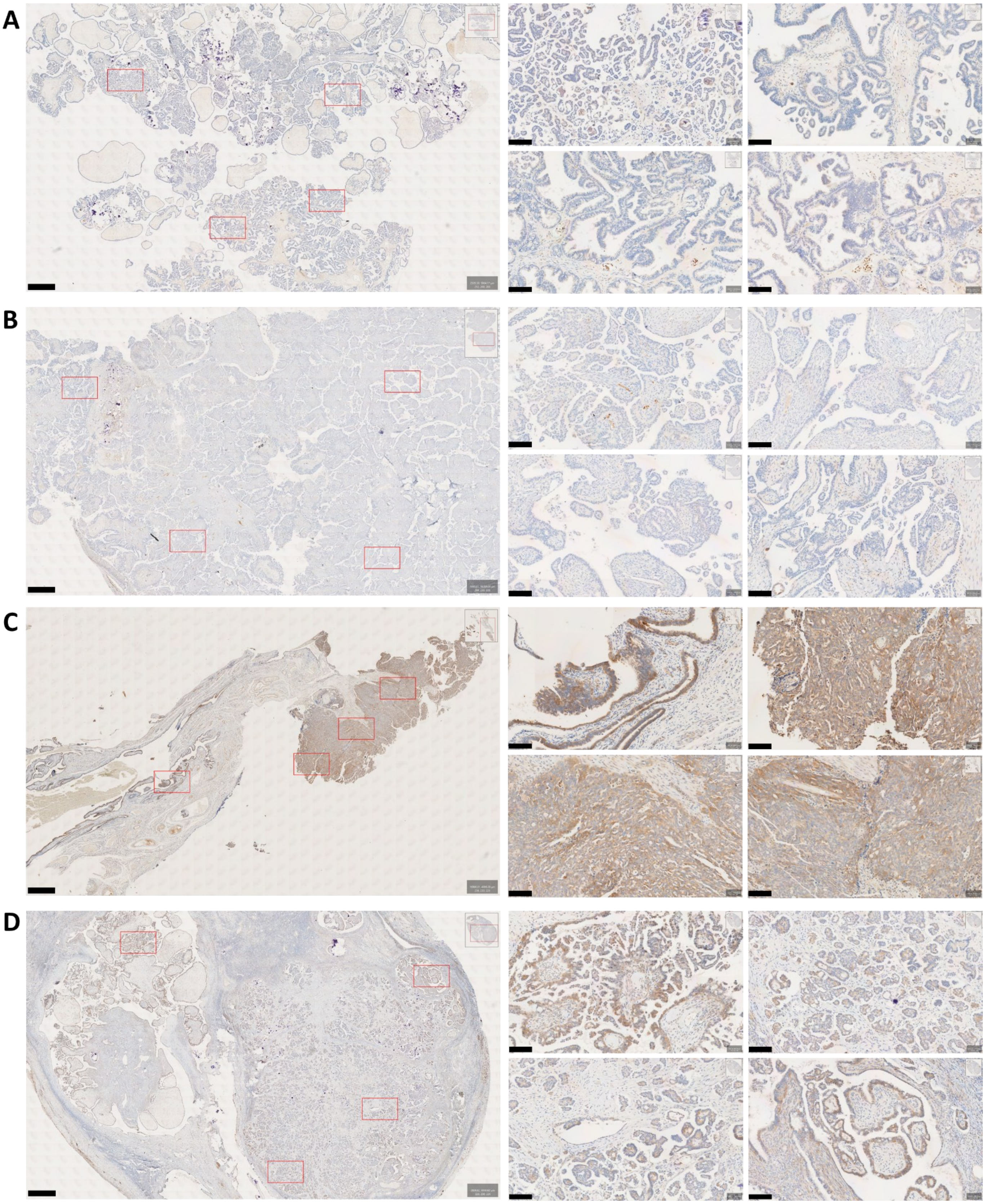

Supplementary Figure S3. Example images from whole-slide phosphorylated ERK (pERK) immunohistochemistry. Scale bars (black) represent 800µm for left hand panels and 50µm on right hand side. A, B, C and D each represent a sample from a different patient included in the study.

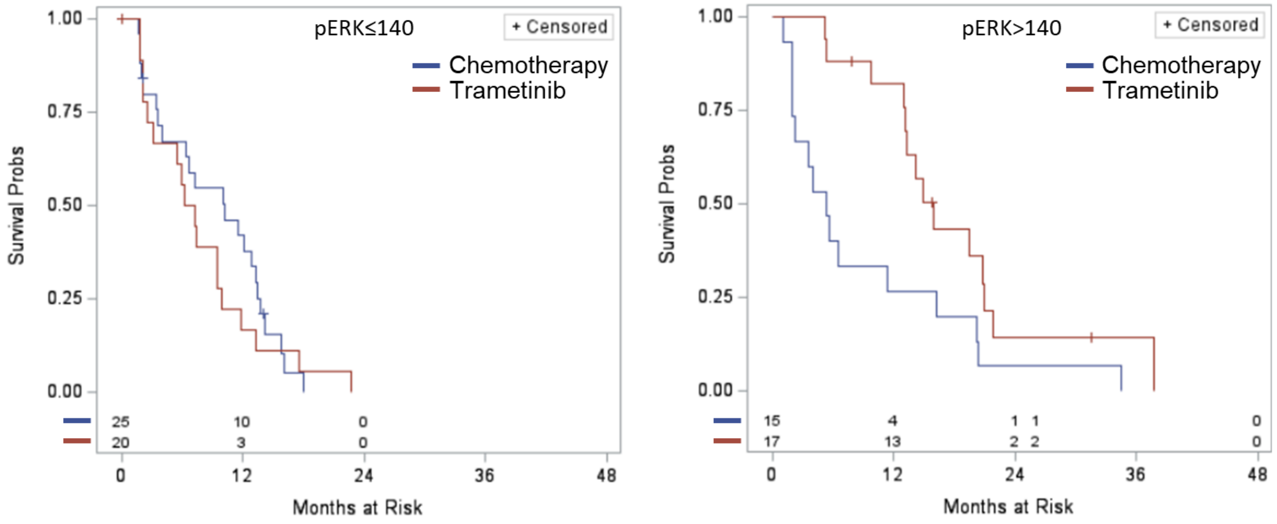

Supplementary Figure S4. Progression-free survival (PFS) of trametinib versus chemotherapy standard of care regimens in individuals with low-pERK tumours (left) and high-pERK tumours (right).

Supplementary tables

Supplementary Table S1. Comparison of translational cohort and total GOG281 trial cohort

		Translational cohort						Total GOG281 trial cohort					
				Randomized RX						Randomized RX			
		All, N=170		SOC, N=85		Trametinib, N=85		All, N=260		SOC, N=130		Trametinib, N=130	
		N	%	N	%	N	%	N	%	N	%	N	%
**Performance status**	**ECOG 0**	122	71.8	63	74.1	59	69.4	186	71.5	93	71.5	93	71.5
	**ECOG 1**	48	28.2	22	25.9	26	30.6	74	28.5	37	28.5	37	28.5
**Stage at diagnosis**	**FIGO I/II**	24	14.1	10	11.8	14	16.5	41	15.8	19	14.6	22	16.9
	**FIGO III**	129	75.9	65	76.5	64	75.3	189	72.7	93	71.5	96	73.8
	**FIGO IV**	17	10.0	10	11.8	7	8.2	30	11.5	18	13.8	12	9.2
**Primary site**	**Ovary**	153	90.0	76	89.4	77	90.6	236	90.8	117	90.0	119	91.5
	**Peritoneum**	17	10.0	9	10.6	8	9.4	24	9.2	13	10.0	11	8.5
**Best response**	**CR**	2	1.2	1	1.2	1	1.2	2	0.8	1	0.8	1	0.8
	**PR**	21	12.4	4	4.7	17	20.0	40	15.4	7	5.4	33	25.4
	**Stable**	113	66.5	60	70.6	53	62.4	169	65.0	92	70.8	77	59.2
	**Progressing**	23	13.5	17	20.0	6	7.1	31	11.9	22	16.9	9	6.9
	**Undetermined**	11	6.5	3	3.5	8	9.4	18	6.9	8	6.2	10	7.7
**Age (median)**	**10yr interval**	50-59		50-59		50-59		50-59		50-59		50-59	

Supplementary Table S2. Demographics of translational research population and subpopulations.

		Translational cohort						pERK cohort						WES cohort					
				Arm						Arm						Arm			
		All, N=170		SOC, N=85		Trametinib, N=85		All, N=148		SOC, N=75		Trametinib, N=73		All, N=134		SOC, N=64		Trametinib, N=70	
		N	%	N	%	N	%	N	%	N	%	N	%	N	%	N	%	N	%
Age (years)	18-29	15	8.8	7	8.2	8	9.4	15	10.1	7	9.3	8	11.0	14	10.4	6	9.4	8	11.4
	30-39	19	11.2	12	14.1	7	8.2	16	10.8	11	14.7	5	6.8	14	10.4	8	12.5	6	8.6
	40-49	33	19.4	18	21.2	15	17.6	30	20.3	17	22.7	13	17.8	29	21.6	14	21.9	15	21.4
	50-59	42	24.7	17	20.0	25	29.4	33	22.3	11	14.7	22	30.1	31	23.1	13	20.3	18	25.7
	60-69	43	25.3	23	27.1	20	23.5	39	26.4	22	29.3	17	23.3	33	24.6	16	25.0	17	24.3
	≥70	18	10.6	8	9.4	10	5.9	15	10.1	7	9.3	8	11.0	13	9.7	7	10.9	6	8.6
Performance status	ECOG 0	122	71.8	63	74.1	59	69.4	111	75.0	59	78.7	52	71.2	95	70.9	46	71.9	49	70.0
	ECOG 1	48	28.2	22	25.9	26	30.6	37	25.0	16	21.3	21	28.8	39	29.1	18	28.1	21	30.0
Stage at diagnosis	FIGO I/II	24	14.1	10	11.8	14	16.5	20	13.5	8	10.7	12	16.4	16	11.9	5	7.8	11	15.7
	FIGO III	129	75.9	65	76.5	64	75.3	113	76.4	57	76.0	56	76.7	104	77.6	51	79.7	53	75.7
	FIGO IV	17	10.0	10	11.8	7	8.2	15	10.1	10	13.3	5	6.8	14	10.4	8	12.5	6	8.6
Primary site	Ovary	153	90.0	76	89.4	77	90.6	134	90.5	69	92.0	65	89.0	123	91.8	56	87.5	67	95.7
	Peritoneum	17	10.0	9	10.6	8	9.4	14	9.5	6	8.0	8	11.0	11	8.2	8	12.5	3	4.3
Best response	CR	2	1.2	1	1.2	1	1.2	2	1.4	1	1.3	1	1.4	2	1.5	1	1.6	1	1.4
	PR	21	12.4	4	4.7	17	20.0	15	10.1	2	2.7	13	17.8	18	13.4	4	6.3	14	20.0
	Stable	113	66.5	60	70.6	53	62.4	100	67.6	53	70.7	47	64.4	88	65.7	46	71.9	42	60.0
	Progressing	23	13.5	17	20.0	6	7.1	21	14.2	16	21.3	5	6.8	17	12.7	11	17.2	6	8.6
	Undetermined	11	6.5	3	3.5	8	9.4	10	6.8	3	4.0	7	9.6	9	6.7	2	3.1	7	10.0
Survival Status	Alive	93	54.7	43	50.6	50	58.8	81	54.7	40	53.3	41	56.2	73	54.5	32	50.0	41	58.6
	Deceased	77	45.3	42	49.4	35	41.2	67	45.3	35	46.7	32	43.8	61	45.5	32	50.0	29	41.4

pERK, phosphorylated ERK. WES, whole exome sequencing. ECOG, Eastern Cooperative Oncology Group. FIGO, International Federation of Gynaecology and Obstetrics. CR, Complete response. PR, partial response.

Supplementary Table S3. Assessment of tolerance to adjusting threshold for defining pERK-high tumours

			Threshold: 120		Threshold: 140		Threshold: 160	
			**pERK>120**	**pERK≤120**	**pERK>140**	**pERK≤140**	**pERK>160**	**pERK≤160**
**Treatment arm**	**Trametinib**	**N**	42	31	38	35	30	43
	**SOC**	**N**	38	37	34	41	29	46
**PFS comparison**	**Trametinib vs SOC**	**HR, 95% CI**	0.37, 0.22-0.62	0.70, 0.42-1.17	0.33, 0.19-0.57	0.75, 0.46-1.23	0.36, 0.20-0.66	0.56, 0.35, 0.90
**Median PFS**	**Trametinib**	**months, 95% CI**	19.5, 13.0-21.0	7.3, 5.6-11.8	20.1, 14.1-21.2	7.5, 5.5-9.5	19.5, 13.0-21.0	9.0, 5.9-12.7
	**SOC**	**months, 95% CI**	5.6, 3.6-11.0	7.3, 3.5-11.2	5.6, 3.6-11.0	7.3, 3.5-11.2	5.7, 3.6-11.0	6.7, 3.5-11.2

Supplementary Table S4. Variant calls in MAPK-associated genes

Hugo_Symbol	Entrez_Gene_Id	Chromosome	Start_Position	End_Position	Variant_Classification	Reference_Allele	Tumor_Seq_Allele1	Tumor_Seq_Allele2	dbSNP_RS	HGVSc	HGVSp_Short	t_depth	t_ref_count	t_alt_count
ASH1L	55870	chr1	155352757	155352757	Missense_Mutation	G	G	A	rs766745788	c.7330C>T	p.R2444C	99	43	56
ASH1L	55870	chr1	155415804	155415804	Missense_Mutation	C	C	T	rs766422974	c.5948G>A	p.R1983H	110	63	46
ASH1L	55870	chr1	155481420	155481420	Nonsense_Mutation	G	G	A	novel	c.1450C>T	p.R484*	20	11	9
PEA15	8682	chr1	160213167	160213167	Missense_Mutation	C	C	T	novel	c.293C>T	p.T98I	324	201	123
MAP4K2	5871	chr11	64802449	64802449	Missense_Mutation	C	C	G	rs149943726	c.280G>C	p.G94R	201	93	105
PTPN6	5777	chr12	6960709	6960710	Frame_Shift_Ins	-	-	GGGGTGGCCAGAG	rs1189997374	c.1724_1736dup	p.L580Wfs*16	232	123	100
BRAP	8315	chr12	111644369	111644369	Missense_Mutation	T	T	C	rs1467391636	c.1609A>G	p.M537V	360	226	134
P2RX7	5027	chr12	121160949	121160949	Frame_Shift_Del	G	G	-	rs1337879229	c.413del	p.G138Dfs*16	232	109	123
MAP3K9	4293	chr14	70732812	70732812	Missense_Mutation	C	C	T	rs986655970	c.2599G>A	p.D867N	313	186	127
MEF2A	4205	chr15	99712743	99712743	Missense_Mutation	G	G	A	rs770266173	c.1490G>A	p.R497Q	229	129	100
NOD2	64127	chr16	50731788	50731788	Missense_Mutation	T	T	A	rs1451481984	c.3092T>A	p.L1031H	307	177	130
MINK1	50488	chr17	4896740	4896740	Missense_Mutation	C	C	T	rs779185115	c.3842C>T	p.T1281M	187	57	130
MAP2K3	5606	chr17	21304467	21304467	Missense_Mutation	G	G	A	rs377202574	c.610G>A	p.V204M	418	344	74
TAOK1	57551	chr17	29495650	29495650	Nonsense_Mutation	C	C	T	novel	c.922C>T	p.Q308*	97	68	29
NF1	4763	chr17	31219119	31219119	Splice_Site	G	G	A	rs1555612866	c.1641+1G>A	p.X547_splice	64	17	47
NF1	4763	chr17	31223502	31223502	Frame_Shift_Del	A	-	-	.	c.1780del	p.T594Qfs*11	12	1	11
NF1	4763	chr17	31235728	31235728	Nonsense_Mutation	C	C	T	rs199474742	c.3826C>T	p.R1276*	139	67	72
CCL3	0	chr17	36088733	36088733	Missense_Mutation	C	C	T	novel	c.218G>A	p.C73Y	84	45	39
PLVAP	83483	chr19	17377225	17377225	Missense_Mutation	A	A	G	rs780657390	c.64T>C	p.W22R	458	222	236
NPHS1	4868	chr19	35831116	35831116	Missense_Mutation	G	G	A	rs143092783	c.3418C>T	p.R1140C	94	52	42
NPHS1	4868	chr19	35851091	35851091	Splice_Site	T	T	C	rs1555763974	c.398-2A>G	p.X133_splice	126	75	51
SBK2	646643	chr19	55530292	55530292	Missense_Mutation	C	C	A	rs1005330136	c.488G>T	p.C163F	43	25	17
SBK2	646643	chr19	55530293	55530293	Missense_Mutation	A	A	G	rs1035028145	c.487T>C	p.C163R	43	25	17
MAP4K4	9448	chr2	101860894	101860894	Missense_Mutation	T	T	A	rs572481216	c.1774T>A	p.L592M	220	130	90
SHANK3	85358	chr22	50684597	50684597	Missense_Mutation	G	G	A	rs371238756	c.976G>A	p.A326T	175	63	112
RAF1	5894	chr3	12604200	12604200	Missense_Mutation	G	G	A	rs80338796	c.770C>T	p.S257L	123	38	84
TNIK	23043	chr3	171068866	171068866	Missense_Mutation	A	A	T	rs768116642	c.3681T>A	p.D1227E	62	20	42
EPGN	255324	chr4	74309092	74309092	Splice_Site	G	G	A	rs750202285	c.44-1G>A	p.X15_splice	192	106	86
EPGN	255324	chr4	74309092	74309092	Splice_Site	G	G	A	rs750202285	c.44-1G>A	p.X15_splice	210	125	84
RAPGEF2	9693	chr4	159323617	159323617	Missense_Mutation	G	G	C	novel	c.1131G>C	p.Q377H	42	28	14
MAP3K1	4214	chr5	56875237	56875237	Missense_Mutation	T	T	C	rs747821377	c.1892T>C	p.V631A	136	62	74
MAP3K1	4214	chr5	56882854	56882854	Missense_Mutation	T	T	G	.	c.3654T>G	p.I1218M	124	57	67
MAPK13	5603	chr6	36138941	36138941	Missense_Mutation	G	G	A	rs746162553	c.904G>A	p.A302T	282	147	135

MAP3K4	4216	chr6	161070780	161070780	Missense_Mutation	T	T	G	novel	c.1880T>G	p.I627R	119	84	35
CD36	948	chr7	80663104	80663104	Missense_Mutation	T	T	C	rs779975352	c.544T>C	p.Y182H	206	114	92
USP17L2	645836	chr8	12137436	12137436	Missense_Mutation	T	T	A	rs772968300	c.1325A>T	p.E442V	698	567	129
USP17L2	645836	chr8	12138249	12138249	Missense_Mutation	G	G	T	rs774321188	c.512C>A	p.A171D	183	141	42
USP17L2	645836	chr8	12138525	12138525	Missense_Mutation	C	C	T	rs201032518	c.236G>A	p.G79E	472	418	52
USP17L2	645836	chr8	12138525	12138525	Missense_Mutation	C	T	T	rs201032518	c.236G>A	p.G79E	40	0	40
USP17L2	645836	chr8	12138525	12138525	Missense_Mutation	C	C	T	rs201032518	c.236G>A	p.G79E	930	817	113
SORBS3	10174	chr8	22571027	22571027	Missense_Mutation	C	C	T	rs149782492	c.1549C>T	p.R517W	438	231	207
SORBS3	10174	chr8	22571222	22571222	Splice_Site	G	G	A	novel	c.1743+1G>A	p.X581_splice	187	163	24
NPR2	4882	chr9	35802545	35802545	Missense_Mutation	G	G	A	rs746601736	c.1753G>A	p.A585T	129	72	57
PTPN3	5774	chr9	109381663	109381663	Missense_Mutation	C	C	T	novel	c.2653G>A	p.V885M	218	119	99
MAP3K15	389840	chrX	19380239	19380239	Missense_Mutation	C	C	T	rs777273513	c.2470G>A	p.G824R	114	59	55
ARAF	369	chrX	47570897	47570897	Missense_Mutation	G	G	A	rs377675069	c.1580G>A	p.R527H	246	187	59
NRK	203447	chrX	105940026	105940026	Missense_Mutation	A	A	G	novel	c.3952A>G	p.S1318G	48	27	18

Supplementary Table S5. Chromosome arm copy number calls in whole exome sequenced cases. -1, copy number loss; 0, no loss/gain; 1, copy number gain.

WES Case ID	1p	1q	2p	2q	3p	3q	4p	4q	5p	5q	6p	6q	7p	7q	8p	8q	9p	9q	10p	10q	11p	11q	12p	12q	13q	14q	15q	16p	16q	17p	17q	18p	18q	19p	19q	20p	20q	21q	22q	Xp	Xq
1	-1	1	0	0	0	0	0	0	0	0	0	0	0	0	0	0	-1	-1	0	0	0	0	0	0	0	0	0	0	0	0	0	-1	-1	1	0	0	0	0	0	0	0
2	-1	1	0	0	-1	1	0	0	0	0	0	0	0	0	0	0	0	0	0	0	0	0	0	0	0	0	0	0	0	0	0	-1	-1	1	0	0	0	0	0	0	0
3	0	0	0	0	0	0	0	0	0	0	0	-1	0	0	0	0	0	0	0	0	0	0	0	0	0	0	0	0	0	0	0	0	0	0	0	0	0	0	0	0	0
4	0	0	0	0	0	0	0	0	0	0	0	0	0	0	1	0	0	0	0	0	0	0	0	0	0	0	0	0	0	0	0	0	0	0	0	0	0	0	0	0	0
5	-1	1	0	0	0	0	0	-1	0	0	0	0	0	0	0	0	-1	0	0	0	0	0	0	0	0	0	0	0	0	0	0	0	0	1	0	0	0	0	-1	0	0
6	0	0	0	0	0	0	0	0	0	0	0	0	0	0	0	0	0	0	0	0	0	0	0	0	0	0	0	0	0	0	0	0	-1	0	0	0	0	0	0	0	-1
7	0	0	0	0	0	0	0	0	0	0	0	0	0	0	0	0	0	0	0	0	0	0	0	0	0	0	0	0	0	0	0	0	0	0	0	0	0	0	0	0	0
8	-1	0	0	0	0	0	0	0	0	0	0	0	0	0	0	0	-1	-1	0	0	0	0	0	0	0	0	0	0	0	0	0	0	0	0	0	0	0	-1	-1	0	0
9	-1	0	0	0	0	0	0	0	0	0	0	0	0	0	1	1	-1	0	0	0	0	0	0	0	0	0	0	0	0	0	0	-1	-1	0	0	1	1	0	-1	0	0
10	0	0	0	0	0	0	0	0	0	0	0	0	0	0	0	0	0	0	0	0	0	0	0	0	0	0	0	0	0	0	0	0	0	0	0	0	0	0	0	0	0
11	-1	1	0	0	0	0	-1	-1	0	0	0	0	0	0	0	0	-1	-1	0	0	0	0	0	0	-1	0	0	-1	-1	0	0	0	0	1	0	0	0	0	-1	-1	-1
12	-1	0	0	0	0	0	0	0	0	0	0	0	0	0	1	1	0	0	0	0	0	0	0	0	0	0	0	0	0	0	0	0	0	0	0	0	0	0	-1	0	0
13	-1	1	0	0	0	0	0	0	0	0	1	-1	0	0	0	0	0	0	0	0	0	0	1	1	0	0	0	0	0	0	0	0	0	0	0	0	1	0	0	0	-1
14	0	0	0	0	0	0	0	0	0	0	0	0	0	0	0	0	0	0	0	0	0	0	0	0	0	0	0	0	0	0	0	0	0	1	0	0	0	0	0	0	-1
15	-1	1	0	0	0	0	0	0	0	0	0	0	0	0	0	0	-1	0	0	0	0	0	0	0	0	0	0	0	0	0	0	0	0	1	0	0	0	0	0	0	0
16	-1	1	0	0	0	0	0	0	0	0	0	0	0	0	1	1	-1	-1	0	0	0	0	0	0	0	0	0	0	0	0	0	-1	-1	0	0	0	0	0	0	0	0
17	-1	1	0	0	0	0	0	0	0	0	0	0	0	0	0	0	-1	0	0	0	0	0	0	0	0	0	0	0	0	0	0	0	0	0	0	0	0	0	-1	0	0
18	0	0	0	0	0	0	0	0	0	0	0	0	0	0	0	0	0	0	0	0	0	0	0	0	0	0	0	0	0	0	0	0	0	0	0	0	0	0	0	0	0
19	-1	0	0	0	-1	1	0	0	0	0	0	0	0	0	0	0	-1	-1	0	0	0	0	0	0	0	0	0	0	0	0	0	0	0	0	0	0	0	0	0	0	0
20	0	0	0	0	0	0	0	0	0	0	0	-1	0	0	0	0	-1	-1	0	0	0	0	0	0	0	-1	0	0	0	0	0	0	0	0	0	0	0	0	-1	0	0
21	0	0	0	0	0	0	0	0	0	0	0	0	0	0	0	0	0	0	0	0	0	0	0	0	0	0	0	0	0	0	0	0	0	0	0	0	0	0	0	-1	-1
22	-1	0	0	0	0	0	0	0	0	0	0	0	0	0	0	0	-1	0	0	0	0	0	1	1	0	0	-1	0	0	0	0	-1	-1	0	0	0	0	0	0	0	0
23	0	0	0	0	0	1	0	0	0	-1	0	-1	0	0	0	1	0	1	0	-1	0	0	1	0	0	0	0	0	0	-1	0	0	0	0	0	-1	0	0	-1	0	-1
24	0	1	0	0	0	0	0	0	0	0	0	0	1	1	0	0	0	0	0	0	0	0	0	0	0	0	0	0	-1	0	0	0	0	0	0	0	0	0	0	-1	-1
25	-1	1	0	0	0	0	-1	-1	0	0	0	0	0	0	0	0	0	0	0	0	-1	0	0	0	0	0	0	0	0	0	0	0	0	0	0	0	0	0	0	0	0
26	-1	1	0	0	0	0	0	0	0	0	0	0	0	0	0	0	0	0	0	0	0	0	0	0	0	0	0	0	0	0	0	0	-1	0	0	0	0	0	0	-1	0
27	0	1	0	0	0	0	0	0	0	0	0	0	0	0	0	0	0	0	0	0	0	0	0	0	0	0	0	0	0	0	0	0	0	0	0	0	0	0	0	0	0
28	-1	1	0	0	0	0	0	0	0	0	0	0	0	0	0	0	0	0	0	0	0	0	0	0	0	0	0	0	0	0	0	0	0	0	0	1	1	0	-1	0	0
29	-1	1	0	0	0	0	-1	0	0	0	0	-1	0	0	0	0	-1	-1	0	0	0	0	0	0	0	0	0	0	0	0	0	0	0	0	0	0	0	0	0	0	0
30	0	0	0	0	0	0	0	0	0	0	0	0	0	0	1	1	-1	-1	0	0	0	0	0	0	0	1	0	0	0	0	0	0	0	0	0	0	1	0	-1	0	0
31	0	0	0	0	0	0	0	0	0	0	0	-1	0	0	1	1	-1	0	0	0	0	0	0	0	0	0	0	0	0	0	0	0	0	0	0	0	0	0	0	0	0
32	-1	0	0	0	0	0	0	0	0	0	0	0	0	0	0	0	0	0	0	0	0	0	0	0	0	0	0	0	0	0	0	0	0	0	0	0	0	0	0	0	0
33	0	1	1	0	0	1	0	0	1	0	0	-1	1	0	0	0	0	0	1	-1	-1	0	0	-1	0	-1	-1	0	1	-1	0	0	0	1	-1	0	1	0	-1	-1	-1

**34**	0	0	0	0	0	0	0	0	0	0	0	0	1	1	0	0	0	0	0	0	0	0	0	0	0	0	0	0	0	0	0	0	0	0	0	0	0	0	0	0	0
**35**	-1	1	0	0	0	0	0	0	0	0	0	0	0	0	0	0	0	0	0	0	0	0	0	0	0	0	0	0	0	0	0	0	0	0	0	0	0	0	0	0	0
**36**	-1	1	0	0	0	0	0	0	0	0	0	0	0	0	0	0	0	0	0	0	0	0	0	0	0	0	0	0	0	0	0	0	0	0	0	0	0	0	0	0	0
**37**	0	1	0	0	0	0	0	0	0	0	0	0	0	0	0	0	0	0	0	0	0	0	0	0	0	0	0	0	0	0	0	0	0	0	0	0	0	0	0	-1	-1
**38**	0	0	0	0	0	0	0	0	0	0	0	0	0	0	0	0	-1	0	0	0	0	0	0	0	0	0	0	0	0	0	0	0	0	0	0	0	0	0	0	0	0
**39**	-1	0	0	0	0	0	0	0	0	0	0	0	0	0	0	0	0	0	0	0	0	0	0	0	0	0	-1	0	0	0	0	0	0	1	0	0	0	0	-1	0	0
**40**	0	0	0	0	0	0	0	0	0	0	0	0	0	0	1	1	0	0	0	0	0	0	0	0	0	0	0	0	0	0	0	0	0	0	0	0	0	0	0	0	0
**41**	-1	1	0	0	0	0	-1	-1	0	0	0	0	0	0	0	0	-1	-1	0	0	0	0	0	0	0	0	-1	0	0	0	0	-1	-1	0	0	1	1	0	-1	-1	-1
**42**	-1	0	0	0	0	0	0	0	0	0	0	-1	0	0	1	0	-1	-1	0	0	0	0	0	0	0	0	0	0	0	0	0	-1	-1	0	0	0	0	0	0	-1	0
**43**	-1	1	0	0	0	0	0	0	0	0	0	0	0	0	0	0	-1	1	0	0	0	0	0	0	0	0	0	0	0	0	0	0	0	0	0	0	0	0	0	0	0
**44**	-1	1	0	0	0	0	-1	-1	0	0	0	0	0	0	0	0	0	0	0	0	0	0	0	0	0	0	0	0	0	0	0	0	0	1	0	1	0	0	0	0	0
**45**	0	1	0	0	0	0	0	0	0	0	0	0	0	0	0	0	0	0	0	0	0	0	0	0	0	0	0	0	0	0	0	0	0	0	0	0	0	0	0	0	0
**46**	0	0	0	0	0	0	0	0	0	0	0	-1	0	0	0	0	0	0	0	0	0	0	0	0	0	0	0	0	0	0	0	0	0	0	0	0	0	0	0	-1	-1
**47**	0	0	0	0	0	1	0	0	0	0	0	0	0	0	0	0	0	0	0	0	0	0	0	0	0	0	0	0	0	0	0	0	0	0	0	0	0	0	0	0	0
**48**	0	0	0	0	0	0	-1	0	0	0	0	0	0	0	0	0	-1	0	0	0	0	0	0	0	0	0	0	0	0	0	0	0	-1	0	0	0	0	0	0	-1	-1
**49**	0	0	0	0	0	0	0	0	0	0	0	0	0	0	-1	1	0	0	0	0	0	0	1	1	0	0	0	0	0	0	0	0	0	-1	-1	0	0	0	0	0	0
**50**	0	0	0	0	0	0	0	0	0	0	0	0	0	0	0	0	0	0	0	0	0	0	0	0	0	0	0	0	0	0	0	0	0	0	0	0	0	0	0	0	0
**51**	0	0	0	0	0	0	0	0	0	0	0	0	0	0	0	0	0	0	0	0	0	0	0	0	0	0	0	0	0	0	0	-1	-1	0	0	0	0	0	0	0	0
**52**	-1	1	0	0	0	0	0	0	0	0	0	0	0	0	0	0	0	0	0	0	0	0	0	0	0	0	0	0	0	0	0	0	0	0	0	0	0	0	0	0	0
**53**	0	1	0	0	0	0	0	0	0	0	0	-1	0	0	1	1	0	0	0	0	0	0	1	1	0	1	0	0	0	0	0	0	0	0	0	0	0	0	0	-1	0
**54**	0	0	0	0	0	0	0	0	0	0	0	0	0	0	1	1	0	0	0	0	0	0	0	0	0	0	0	0	0	0	0	0	0	0	0	0	0	0	0	0	0
**55**	0	0	0	0	0	0	0	0	0	0	0	0	0	0	0	0	0	0	0	0	0	0	0	0	0	0	0	0	0	0	0	0	0	0	0	0	0	0	0	0	0
**56**	-1	0	0	0	0	0	0	0	0	0	0	0	0	0	0	0	0	0	0	0	0	0	0	0	0	0	0	0	0	0	0	0	0	0	-1	0	0	0	-1	0	0
**57**	0	0	0	0	0	0	0	0	0	0	0	0	0	0	0	0	0	0	0	0	0	0	0	0	0	0	0	0	0	0	0	0	0	0	0	0	0	0	0	0	0
**58**	0	1	0	0	0	0	0	0	-1	-1	0	0	0	0	1	1	-1	0	0	0	0	0	0	0	1	1	0	0	0	0	0	0	-1	1	0	0	0	0	0	0	0
**59**	-1	1	0	0	0	0	0	0	0	0	-1	-1	0	1	-1	1	0	0	0	0	0	0	1	1	0	0	0	-1	-1	0	0	1	0	0	0	0	0	0	-1	0	0
**60**	0	0	0	0	0	0	0	0	0	0	0	0	0	0	0	0	0	0	0	0	0	0	0	0	0	0	0	0	0	0	0	0	0	0	0	0	0	0	0	0	-1
**61**	-1	1	0	0	0	0	0	0	0	0	0	0	0	0	0	0	0	0	0	0	0	0	0	0	0	0	0	0	0	0	0	0	-1	0	0	0	1	0	-1	0	0
**62**	0	0	0	0	0	0	0	0	0	0	0	0	0	0	0	0	0	0	0	0	0	0	0	0	0	0	-1	0	0	0	0	0	0	1	0	0	0	0	-1	0	0
**63**	-1	1	0	0	0	0	0	0	0	0	0	0	0	0	0	0	0	0	0	0	0	0	0	0	0	0	0	0	0	0	0	0	0	0	0	0	0	0	-1	0	0
**64**	-1	0	0	0	0	0	0	0	0	0	-1	-1	0	0	0	0	-1	0	0	0	0	0	1	1	0	0	-1	0	0	0	0	-1	-1	0	0	1	1	0	0	-1	-1
**65**	-1	0	0	0	0	0	0	0	0	0	0	0	0	0	0	0	0	0	0	0	0	0	0	0	0	0	0	0	0	-1	1	0	0	0	-1	0	0	0	0	0	0
**66**	0	0	0	0	0	0	0	0	0	0	0	0	0	0	0	0	-1	0	0	0	0	0	0	0	0	-1	0	0	-1	-1	0	-1	-1	0	0	0	0	0	-1	0	0
**67**	-1	0	0	0	0	0	0	0	0	0	0	0	0	0	0	0	-1	0	0	0	0	0	0	0	0	0	0	0	0	0	0	0	0	0	0	0	0	0	-1	0	0
**68**	-1	1	0	0	0	0	0	0	0	0	0	0	0	0	1	1	0	0	0	0	0	0	0	0	0	0	0	0	0	0	0	-1	-1	1	0	1	1	-1	0	0	-1
**69**	0	0	0	0	0	0	0	0	0	0	0	0	0	0	0	0	0	0	0	0	0	0	0	0	0	0	0	0	0	0	0	0	0	0	0	0	0	0	0	0	0

**70**	0	0	0	0	0	0	0	0	0	0	0	0	0	0	1	1	0	0	0	0	0	0	0	0	0	0	0	0	0	0	0	0	0	1	0	0	0	0	0	0	0
**71**	0	0	0	0	0	0	0	0	0	0	1	-1	0	0	0	0	0	0	1	0	0	0	0	0	0	0	0	0	0	0	0	0	0	0	0	0	0	0	0	0	0
**72**	0	0	0	0	0	0	0	0	0	0	0	0	0	0	0	0	0	0	0	0	0	0	0	0	0	0	0	0	0	0	0	0	0	0	0	0	0	0	0	0	0
**73**	0	0	0	0	0	0	0	0	0	0	0	0	0	0	0	0	0	0	0	0	-1	0	0	0	0	0	0	0	0	0	0	0	0	0	0	0	0	0	0	0	0
**74**	-1	1	0	0	-1	0	0	0	0	0	0	0	0	0	0	0	0	0	0	0	0	0	0	0	0	0	0	0	0	0	0	0	0	0	0	0	0	-1	0	0	0
**75**	-1	1	0	0	0	1	0	0	0	0	0	0	0	0	0	0	0	0	1	0	0	0	1	1	0	0	0	0	0	0	0	0	0	0	0	0	1	0	0	0	-1
**76**	0	0	0	0	0	0	0	0	0	0	0	0	0	0	0	0	0	0	0	0	0	0	0	0	0	0	0	0	0	0	0	0	0	0	0	0	0	0	0	0	0
**77**	-1	1	0	0	0	0	0	0	0	0	0	0	0	0	-1	1	0	0	0	0	0	0	0	0	0	0	-1	0	0	0	0	0	0	1	0	1	1	0	-1	0	0
**78**	-1	0	0	0	0	0	0	0	0	0	0	0	0	0	0	0	0	0	0	0	0	0	0	0	0	0	0	0	0	0	0	0	0	1	0	0	0	0	0	-1	-1
**79**	-1	1	0	0	0	0	0	0	0	0	0	0	0	0	0	0	-1	0	0	0	0	0	0	0	0	0	0	0	0	0	0	0	0	1	0	0	0	0	-1	0	0
**80**	0	0	0	0	0	0	0	0	0	0	0	0	0	0	0	0	0	0	0	0	0	0	0	0	0	0	0	0	0	0	0	0	0	0	0	0	0	0	0	0	0
**81**	-1	0	0	0	0	0	0	0	0	0	0	0	0	0	0	0	0	0	0	0	-1	0	0	0	0	0	0	0	0	0	0	0	0	0	0	0	0	0	0	0	0
**82**	-1	0	0	0	0	0	0	0	0	0	0	0	0	0	0	0	-1	0	0	0	0	0	0	0	0	0	0	0	0	0	1	-1	-1	0	0	0	0	-1	0	0	-1
**83**	0	0	0	0	0	0	0	0	0	0	0	0	0	0	0	0	0	0	0	0	0	0	0	0	0	0	0	0	-1	0	0	0	0	0	0	0	0	0	0	0	0
**84**	0	0	0	0	0	0	0	0	0	0	0	0	0	0	-1	0	0	0	0	0	0	0	0	0	0	0	0	0	-1	0	0	-1	-1	0	-1	0	0	0	-1	-1	-1
**85**	0	0	0	0	0	0	0	0	0	0	0	-1	0	0	1	1	-1	0	0	0	0	0	1	1	0	1	0	0	0	0	0	0	0	0	0	1	1	0	0	-1	-1
**86**	-1	1	0	0	-1	1	-1	-1	0	0	0	0	0	0	0	0	0	0	0	0	0	0	1	1	0	0	0	0	0	0	0	0	0	1	-1	0	0	0	-1	0	0
**87**	0	0	0	0	0	0	0	0	0	0	0	0	0	0	0	0	0	0	0	0	0	0	0	0	0	0	0	0	0	0	0	0	0	1	0	0	0	0	0	0	0
**88**	0	0	0	0	0	0	0	0	0	0	0	0	0	0	0	0	0	0	0	0	0	0	0	0	0	0	0	1	0	0	0	0	0	1	0	0	0	0	0	0	-1
**89**	0	0	0	0	0	0	0	0	0	0	0	0	0	0	0	0	0	0	0	0	0	0	0	0	0	0	0	0	0	0	0	0	0	0	0	0	0	0	0	0	0
**90**	-1	1	0	0	0	0	0	0	0	0	0	0	0	0	0	0	0	0	0	0	0	0	0	0	0	0	0	0	0	0	0	0	0	1	0	0	1	0	-1	-1	-1
**91**	-1	1	0	0	0	0	0	0	0	0	0	0	0	0	0	0	0	0	0	0	0	0	1	1	0	0	0	0	0	0	0	0	0	0	0	0	0	0	0	0	0
**92**	0	0	0	0	0	0	0	0	0	0	0	0	0	0	0	0	-1	0	0	0	0	0	0	0	0	0	0	0	0	0	0	0	0	1	0	0	0	0	0	-1	-1
**93**	-1	1	0	0	0	0	0	0	0	0	0	0	0	0	0	0	0	0	0	0	0	0	1	1	0	0	-1	0	0	0	0	0	0	0	0	0	0	0	0	0	0
**94**	0	1	0	0	0	0	0	0	1	0	0	-1	0	0	0	0	0	0	0	0	0	0	0	0	0	0	0	0	0	0	0	0	0	1	0	0	0	0	0	-1	-1
**95**	-1	1	0	0	0	0	0	0	0	0	0	0	0	0	0	0	0	0	0	0	0	0	0	0	0	0	0	0	0	0	0	0	0	0	0	0	0	0	0	0	0
**96**	0	0	0	0	0	0	0	0	0	0	0	0	0	0	0	0	0	0	0	0	0	0	0	0	0	0	0	0	0	0	0	0	0	1	0	0	0	0	0	0	0
**97**	-1	0	0	0	0	0	0	0	0	0	0	0	0	0	0	0	0	0	0	0	0	0	0	0	0	0	0	0	0	0	0	0	0	0	0	0	0	0	-1	0	0
**98**	-1	0	0	0	0	0	-1	-1	0	0	0	0	1	1	0	0	0	0	0	0	0	0	0	0	0	0	0	0	0	0	0	-1	-1	1	0	0	0	0	0	-1	-1
**99**	0	0	0	0	0	0	0	0	0	0	0	-1	0	0	0	0	0	0	0	0	0	0	0	0	0	0	-1	0	0	-1	0	0	0	1	0	0	0	0	-1	0	0
**100**	0	0	0	0	0	0	0	0	0	0	0	0	0	0	0	0	0	0	0	0	0	0	0	0	0	0	0	0	0	0	0	0	0	1	0	0	0	0	0	0	0
**101**	-1	1	0	0	0	0	0	0	0	0	0	0	0	0	0	0	0	0	0	0	0	0	0	0	0	0	0	0	0	0	0	0	0	1	0	0	0	0	0	0	0
**102**	0	1	0	0	0	0	0	0	0	0	0	0	0	0	0	0	-1	0	0	0	-1	0	0	0	0	0	0	0	0	0	0	0	0	1	0	0	0	0	0	0	0
**103**	-1	1	0	0	0	0	0	0	0	0	1	-1	0	0	0	0	-1	-1	0	0	0	0	0	0	0	0	0	0	0	0	0	0	0	1	1	0	1	0	0	0	0
**104**	-1	0	0	0	0	0	0	0	0	0	0	0	0	0	0	0	-1	1	0	0	0	0	0	0	0	0	0	1	0	0	0	-1	-1	1	0	0	0	0	-1	0	0
**105**	0	0	0	0	0	0	0	0	0	0	0	0	0	0	-1	0	0	0	0	0	0	0	0	0	0	0	0	0	0	0	0	0	0	0	0	0	0	0	0	0	0

**106**	-1	1	0	0	0	0	-1	-1	0	0	0	0	0	1	0	0	0	0	0	0	0	0	0	0	0	1	0	0	0	0	0	0	0	1	0	0	0	0	0	0	0
**107**	-1	1	0	0	0	0	0	0	0	0	0	0	0	0	0	0	0	0	0	0	0	0	0	0	0	0	0	0	0	0	0	0	0	1	0	0	0	0	-1	0	0
**108**	-1	1	0	0	0	0	0	0	0	0	0	0	0	0	-1	1	0	0	0	0	0	0	0	0	0	0	0	0	0	0	0	0	0	1	0	0	0	0	0	0	0
**109**	0	0	0	0	0	0	0	0	0	0	0	0	0	0	0	0	0	0	0	0	0	0	0	0	0	0	0	0	0	0	0	0	0	0	0	0	0	0	0	0	0
**110**	-1	0	0	0	0	0	0	0	0	0	0	0	0	0	0	0	0	0	0	0	0	0	0	0	0	0	0	0	0	0	0	0	0	1	0	0	0	0	0	0	0
**111**	0	0	0	0	0	0	0	0	0	0	0	0	0	0	0	0	0	0	0	0	0	0	0	0	0	0	0	0	0	0	0	0	0	0	0	0	0	0	0	0	0
**112**	0	0	0	0	0	0	0	0	0	0	0	0	0	0	0	0	0	0	0	0	0	0	0	0	0	0	0	0	0	0	0	0	0	1	0	0	0	0	0	0	-1
**113**	-1	0	0	0	0	0	0	0	0	0	0	0	0	0	0	0	0	0	0	0	0	0	0	0	0	0	0	0	0	0	0	0	0	1	0	0	0	0	0	0	0
**114**	0	-1	-1	-1	0	0	0	0	1	1	-1	-1	1	1	1	1	-1	-1	0	0	-1	-1	1	1	-1	0	1	1	0	0	0	-1	-1	0	0	0	0	0	0	-1	-1
**115**	0	0	0	0	0	0	0	0	0	0	0	0	0	0	0	0	-1	-1	0	0	0	0	1	1	0	0	0	0	-1	0	0	0	0	0	-1	0	0	0	-1	-1	-1
**116**	0	1	0	0	0	0	0	0	0	0	0	0	0	0	0	0	-1	0	0	0	0	0	1	0	0	0	0	0	-1	0	0	0	0	0	0	0	0	1	0	0	-1
**117**	0	0	0	0	0	0	0	0	0	0	0	-1	0	0	0	1	0	0	0	0	0	0	0	0	0	0	0	0	0	0	0	0	0	0	-1	0	0	0	-1	0	0
**118**	0	0	0	0	0	0	0	0	0	0	0	0	0	0	0	0	0	0	0	0	0	0	0	0	0	0	0	0	0	0	0	0	0	0	0	0	0	0	0	0	0
**119**	-1	1	0	0	0	0	0	0	0	0	0	0	0	0	0	0	-1	0	0	0	0	0	0	0	0	0	0	0	0	0	0	-1	0	0	0	0	0	0	0	0	0
**120**	0	0	0	0	0	0	0	0	0	0	0	0	0	0	0	0	0	0	0	0	0	0	0	0	0	0	0	0	0	0	0	0	0	0	0	0	0	0	0	0	0
**121**	-1	1	0	0	0	0	0	0	0	0	0	0	0	0	0	0	-1	-1	0	0	0	0	0	0	0	0	0	0	0	0	0	0	0	0	0	0	0	0	-1	-1	-1
**122**	-1	0	0	0	0	1	0	0	0	0	0	0	0	0	0	0	0	0	0	0	0	0	0	0	0	0	0	0	-1	0	0	0	0	0	0	0	0	0	0	0	-1
**123**	0	0	0	0	0	0	0	0	0	0	0	0	0	0	1	0	0	0	0	0	0	0	1	1	0	0	0	0	0	0	0	0	0	0	0	0	0	0	0	0	0
**124**	-1	0	0	0	0	0	0	0	0	0	0	0	0	0	0	0	0	0	0	0	0	0	0	0	0	0	0	0	0	0	0	0	0	0	0	0	0	0	0	0	0
**125**	0	1	0	0	0	0	0	0	0	0	0	0	0	0	0	0	-1	0	0	0	0	0	0	0	0	0	0	1	1	0	0	0	0	0	0	0	0	0	0	-1	-1
**126**	-1	1	0	0	0	0	0	0	0	0	1	-1	0	0	0	0	-1	1	0	0	0	0	0	0	0	0	0	0	0	0	0	0	0	0	0	0	0	-1	-1	0	0
**127**	-1	1	1	0	0	0	0	-1	0	0	0	0	0	0	1	1	0	0	0	0	0	0	0	0	0	0	0	0	0	0	0	0	0	0	0	1	1	0	0	-1	-1
**128**	-1	0	0	0	0	0	-1	0	0	0	0	0	0	0	0	0	0	0	0	0	0	0	0	0	0	0	0	0	0	0	0	0	0	0	0	0	0	0	0	-1	-1
**129**	-1	0	0	0	0	0	0	0	0	0	0	0	0	0	1	1	0	0	0	0	0	0	0	0	0	1	0	0	0	0	0	0	0	0	0	0	0	0	0	-1	-1
**130**	0	0	0	0	0	0	0	0	0	0	0	0	0	0	0	0	0	0	0	0	0	0	0	0	0	0	0	0	0	0	0	0	0	0	0	0	0	0	0	0	0
**131**	-1	0	0	0	0	0	0	0	0	0	0	0	0	0	0	0	-1	-1	0	0	0	0	0	0	0	0	0	0	0	0	0	0	0	1	0	0	0	-1	-1	0	0
**132**	-1	0	0	0	0	0	0	0	0	0	0	0	0	0	1	1	0	0	0	0	0	0	1	1	0	0	0	0	0	0	0	0	0	0	0	0	0	0	0	0	0
**133**	0	0	0	0	0	0	0	0	0	0	0	0	0	0	0	0	0	0	0	0	0	0	0	0	0	0	0	0	0	0	0	0	0	0	0	0	0	0	0	0	0
**134**	0	0	0	0	0	0	0	0	0	0	0	0	0	0	0	0	0	0	0	0	0	0	0	0	0	0	0	0	0	0	0	0	0	0	0	0	0	0	0	0	0

Supplementary Table S6. pERK histoscore across evaluable cases

pERK Case ID	pERK histoscore
A	200
B	180
C	115
D	0
E	270
F	200
G	5
H	5
I	80
J	0
K	40
L	40
M	0
N	240
O	270
P	200
Q	70
R	80
S	40
T	0
U	80
V	100
W	50
X	50
Y	40
Z	0
AA	80
AB	0
AC	20
AD	120
AE	75
AF	240
AG	120
AH	10
AI	1
AJ	30
AK	30
AL	240
AM	260
AN	1
AO	200
AP	160
AQ	270
AR	210
AS	20
AT	180

**AU**	300
**AV**	100
**AW**	120
**AX**	270
**AY**	270
**AZ**	300
**BA**	300
**BB**	270
**BC**	120
**BD**	120
**BE**	150
**BF**	300
**BG**	160
**BH**	105
**BI**	140
**BJ**	105
**BK**	60
**BL**	255
**BM**	80
**BN**	210
**BO**	55
**BP**	90
**BQ**	225
**BR**	195
**BS**	270
**BT**	285
**BU**	180
**BV**	0
**BW**	300
**BX**	45
**BY**	140
**BZ**	270
**CA**	120
**CB**	240
**CC**	20
**CD**	285
**CE**	255
**CF**	270
**CG**	45
**CH**	180
**CI**	120
**CJ**	90
**CK**	195
**CL**	270
**CM**	165
**CN**	150
**CO**	70
**CP**	60
**CQ**	150

**CR**	60
**CS**	210
**CT**	160
**CU**	195
**CV**	130
**CW**	225
**CX**	40
**CY**	140
**CZ**	225
**DA**	60
**DB**	120
**DC**	300
**DD**	120
**DE**	150
**DF**	40
**DG**	105
**DH**	135
**DI**	255
**DJ**	90
**DK**	145
**DL**	100
**DM**	150
**DN**	210
**DO**	220
**DP**	55
**DQ**	120
**DR**	225
**DS**	195
**DT**	135
**DU**	285
**DV**	150
**DW**	285
**DX**	220
**DY**	70
**DZ**	140
**EA**	250
**EB**	160
**EC**	210
**ED**	160
**EE**	60
**EF**	240
**EG**	25
**EH**	145
**EI**	180
**EJ**	40
**EK**	70
**EL**	170
**EM**	285
**EN**	70

**EO**	40
**EP**	225
**EQ**	270
**ER**	140
